# Effectiveness assessment of using water environmental microHI to predict the health status of wild fish

**DOI:** 10.3389/fmicb.2023.1293342

**Published:** 2024-01-11

**Authors:** Haile Yang, Jia Zhong, Xiaoqian Leng, Jinming Wu, Peilin Cheng, Li Shen, Jinping Wu, Pengcheng Li, Hao Du

**Affiliations:** Key Laboratory of Freshwater Biodiversity Conservation, Ministry of Agriculture and Rural Affairs, Yangtze River Fisheries Research Institute, Chinese Academy of Fishery Sciences, Wuhan, China

**Keywords:** gut microbiota, environmental microbiota, microbial phenotypes, microbiota health index (microHI), aquatic wildlife, health monitoring

## Abstract

Aquatic wildlife health assessment is critically important for aquatic wildlife conservation. However, the health assessment of aquatic wildlife (especially aquatic wild animals) is difficult and often accompanied by invasive survey activities and delayed observability. As there is growing evidence that aquatic environmental microbiota could impact the health status of aquatic animals by influencing their symbiotic microbiota, we propose a non-invasive method to monitor the health status of wild aquatic animals using the environmental microbiota health index (microHI). However, it is unknown whether this method is effective for different ecotype groups of aquatic wild animals. To answer this question, we took a case study in the middle Yangtze River and studied the water environmental microbiota and fish gut microbiota at the fish community level, population level, and ecotype level. The results showed that the gut microHI of the healthy group was higher than that of the unhealthy group at the community and population levels, and the overall gut microHI was positively correlated with the water environmental microHI, whereas the baseline gut microHI was species-specific. Integrating these variations in four ecotype groups (filter-feeding, scraper-feeding, omnivorous, and carnivorous), only the gut microHI of the carnivorous group positively correlated with water environmental microHI. Alcaligenaceae, Enterobacteriaceae, and Achromobacter were the most abundant groups with health-negative-impacting phenotypes, had high positive correlations between gut sample group and environment sample group, and had significantly higher abundance in unhealthy groups than in healthy groups of carnivorous, filter-feeding, and scraper-feeding ecotypes. Therefore, using water environmental microHI to indicate the health status of wild fish is effective at the community level, is effective just for carnivorous fish at the ecotype level. In the middle Yangtze River, Alcaligenaceae, Enterobacteriaceae (family level), and *Achromobacter* (genus level) were the key water environmental microbial groups that potentially impacted wild fish health status. Of course, more data and research that test the current hypothesis and conclusion are encouraged.

## Introduction

Aquatic environmental microbiota could impact the health status of aquatic organisms by influencing their symbiotic microbiota (Guivier et al., 2020; Sehnal et al., 2021; Yang et al., 2022). It is well known that humans co-evolve with their symbiotic microbiota, which plays a critical role in human health (Adak and Khan, 2019; Fassarella et al., 2021; Reynoso-García et al., 2022). There is a similar situation in aquatic organisms, and the symbiotic microbiota plays a more critical role in aquatic organisms (Cabillon and Lazado, 2019; Guivier et al., 2020; Lindsay et al., 2020; Sehnal et al., 2021; Luna et al., 2022). In aquatic animals, environmental microbiota could strongly influence the symbiotic microbiota in the skin, gut, gills, and so on (Reinhart et al., 2019; Guivier et al., 2020; Kuang et al., 2020; Elsheshtawy et al., 2021; Yang et al., 2022), in which the gut microbiota has relatively more stable community components and structures (Guivier et al., 2020), weaker influences from environmental microbiota (Reinhart et al., 2019), and higher impacts on the host’s health (Sehnal et al., 2021) than other symbiotic microbiota (such as the symbiotic microbiota in skin, gills, and so on). Therefore, the gut microbiota is the key point for studying the processes of aquatic environmental microbiota impacting the health status of aquatic animals.

The health status of aquatic animals may be indicated by environmental microHI (Yang et al., 2023). The health of an individual always refers to the individual not being disturbed or hurt. The health of aquatic animals could be indicated by normal gut microbiota (Sun et al., 2020; Tong et al., 2020) and a low abundance of health-negative-impacting microbes (Qiao et al., 2019; Himani et al., 2020). The microHI (microbiota health index) is an abbreviation of the microbiota health index, which is defined as the average abundance ratio of microbes without three health-negative-impacting microbial phenotypes (Eq. 1) (Yang et al., 2023). These three health-negative-impacting microbial phenotypes, respectively, are potential pathogenicity, mobile element content, and oxidative stress tolerance (Yang et al., 2023). The abundance ratio of microbes with potential pathogenicity indicates the potential risk of disease in the host (Himani et al., 2020). The abundance ratio of microbes that contain mobile elements (such as antibiotic resistance genes) reflects the potential risk of antibiotic pollution (Sáenz et al., 2019) or potential threats to host health (Himani et al., 2020). The abundance ratio of microbes with oxidative stress tolerance indicates the potential risk of host gut tissue damage (Qiao et al., 2019; Solomando et al., 2020). The abundance of microbes with these microbial phenotypes can be predicted and quantified based on high-throughput sequencing technology and the BugBase database (https://bugbase.cs.umn.edu/index.html) (Yang et al., 2023). Previous work identified that an individual with a low microHI gut microbiota is more likely to have a disordered microbiota and is more likely obtained from the water with a low aquatic environmental microHI (Yang et al., 2023). As a disordered microbiota always indicates an unhealthy host (Sun et al., 2020; Tong et al., 2020), the aquatic environmental microHI has the potential to indicate the health status of aquatic animals (Yang et al., 2023).


(1) $$\mathrm{microHI}=\frac{\left[\left(1-P_{m}\right)+\left(1-P_{o}\right)+\left(1-P_{p}\right)\right]}{3}$$

where *microHI* denotes the microbiota health index; $P_{m}$ denotes the abundance ratio (percent) of microbes containing mobile elements; $P_{o}$ denotes the abundance ratio (percent) of microbes with oxidative stress tolerance; and $P_{p}$ denotes the abundance ratio (percent) of microbes with potential pathogenicity (Yang et al., 2023).

There is species variation in the relationship between aquatic environmental microbiota and gut microbiota in aquatic animals (Sun and Xu, 2021; Yang et al., 2022). As the gut microbiota is driven by both host-related deterministic selection and the introduction of neutral microbes from the environment (Ringø et al., 2016; Legrand et al., 2020), and the majority of the introduced gut microbiota is derived from the feeding habits of the host (Yang et al., 2022), the correlation between environmental microbiota and gut microbiota is ecotype-specific and even species-specific (Yang et al., 2022). In a previous work, the possibility of using aquatic environmental microHI to indicate the health status of aquatic animals was preliminarily identified at the community level (Yang et al., 2023). Is there any species or ecotype variation in the effectiveness of using water environmental microHI to indicate the health status of aquatic animals? Based on the fact that the gut microbiota is driven by host-related deterministic selection and the introduction of neutral microbes from the environment (Ringø et al., 2016; Legrand et al., 2020), and that the microHI is calculated based on the microbiota community structure (Yang et al., 2023), we hypothesized that (1) the base value of the gut microHI is species-specific, and (2) the correlation between the water environmental microHI and the gut microHI has species variation and ecotype variation. In other words, we hypothesized that there is effectiveness variation in the use of water environmental microHI to indicate the health status of aquatic wild animals at the population level and ecotype level.

To identify the effectiveness variation, we took a case study to research the water environmental microbiota and fish gut microbiota at the fish community level, population level, and ecotype level. As the third longest river in the world with rich aquatic biodiversity, the Yangtze River has received much attention in wildlife conservation (Chen et al., 2020; Zhang et al., 2020). Proposing a convenient method to monitor and indicate the health status of wildlife in the Yangtze River is urgently needed (Yang et al., 2023). In the current work, we collected a data set of the fish gut microbiota and water environmental microbiota in the middle Yangtze River. Using this data set, we further verified that the water environmental microHI could be used to indicate the health of aquatic animals at the community level. Based on the previous data set and the new data set, we tested the effectiveness of using water environmental microHI to indicate the health of wild fish at the population level and ecotype level, and we identified the key microbial groups that influenced the health status of wild fish. The current study clarifies the effectiveness of using water environmental microHI to indicate the health status of wild fish and proves a non-invasive method to monitor the health status of wild fish without harming the individuals, which would offer a new way of understanding and managing the health status of aquatic wild animals and provide a new tool for the toolkit of aquatic wildlife conservation.

## Materials and methods

### Sample collection

From 27 June 2022 to 14 July 2022, we collected gut samples of adult wild fish (Table 1) and water environmental DNA samples at the Jiang’an section of the middle Yangtze River (N 30°38′52″, E 114°20′58″, located in Wuhan City, Hubei Province, People’s Republic of China), along with a conventional Yangtze River fishery resources investigation. Wild fish were caught using a floating gill net, transported to the laboratory in an ice bath, and then dissected. Approximately 0.5 g of gut sample, including both tissues and contents, was aseptically taken from each individual fish caught. A 1.5 L surface water sample was collected each day (including two transection sample; more details in Supplementary Table S1) using a 1.5 L sterilized bottle (rinsed three times with sampling water) and transported to the laboratory in an ice bath. To obtain eDNA samples, each water sample was filtered using a 0.2-μm membrane filter (JinTeng, Tianjin, PRC) with purified water as a negative control. All samples (including gut samples and eDNA samples) were transported on dry ice and then stored at −80°C until DNA extraction. The sampling procedures in the current study were similar to the previous work (Yang et al., 2023).

**Table 1:** Wild fish species in the current study.

**Group label**	**Species**	**Order**	**Family**	**Ecotype**	**Diet**	**Number of samples**
***L.C.***	*Leiocassis crassilabris*	Siluriformes	Bagridae	carnivorous	Oligochaetes, little fish, small mollusks, shrimp	31
***L.L.***	*Leiocassis longirostris*	Siluriformes	Bagridae	carnivorous	Little fish, shrimp, aquatic insects	41
***P.V.***	*Pelteobagrus vachelli*	Siluriformes	Bagridae	carnivorous	Aquatic insects, shrimp, oligochaetes, little fish, small mollusks	4
***P.N.***	*Pelteobagrus nitidus*	Siluriformes	Bagridae	carnivorous	Aquatic insects, shrimp, oligochaetes, little fish	11
***P.F.***	*Pelteobagrus fulvidraco*	Siluriformes	Bagridae	carnivorous	Aquatic insects, shrimp, little fish	16
***P.T.***	*Pseudobagrus tenuis*	Siluriformes	Bagridae	carnivorous	Aquatic insects, mollusks, oligochaetes, crustaceans, little fish	6
***S.A.***	*Silurus asotus*	Siluriformes	Siluridae	carnivorous	Shrimp, little fish	
***S.M.***	*Silurus meridionalis*	Siluriformes	Siluridae	carnivorous	Shrimp, little fish	2
***H.M.***	*Hypophthalmichthys molitrix*	Cypriniformes	Cyprinidae	filter-feeding	Phytoplankton, zooplankton	4
***A.N.***	*Aristichthys nobilis*	Cypriniformes	Cyprinidae	filter-feeding	Zooplankton, phytoplankton	5
***C.H.***	*Coreius heterodon*	Cypriniformes	Cyprinidae	omnivorous	Small mollusks, fish eggs and larvae, phytoclasts	35
***X.A.***	*Xenocypris argentea*	Cypriniformes	Cyprinidae	scraper-feeding	Bottom-attached algae, phytoclasts	14
***S.C.***	*Siniperca chuatsi*	Perciformes	Serranidae	carnivorous	Fish, shrimp	9
***S.K.***	*Siniperca kneri*	Perciformes	Serranidae	carnivorous	Fish, shrimp	5
***C.B.***	*Coilia brachygnathus*	Clupeiformes	Engraulidae	carnivorous	Little fish, shrimp	31

### Microbiome sequencing

The fish gut microbiota and water environmental microbiota were analyzed using 16S rRNA metabarcoding (primers 338F/806R). The sequencing technical details followed those in the previous work (Yang et al., 2020, 2021). Microbial DNA was extracted from gut samples and water eDNA samples using an E.Z.N.A.® Stool DNA Kit (Omega BioTek, Norcross, GA, United States) according to the manufacturer’s protocols. Then, the final DNA concentration and purity were determined using a NanoDrop 2000 UV–vis spectrophotometer (Thermo Fisher Scientific, Wilmington, United States), and DNA quality was checked by 1% agarose gel electrophoresis. The V3–V4 hypervariable regions of the bacterial 16S rRNA gene were amplified with the primers 338F (5′-ACTCCTACGGGAGGCAGCAG-3′) and 806R (5′-GGACTACHVGGGTWTCTAAT-3′) by using a PCR thermocycler system (GeneAmp 9,700, ABI, United States). The PCRs were performed in triplicate 20-μl mixtures containing 4 μL of 5 × FastPfu Buffer, 2 μL of 2.5 mM dNTPs, 0.8 μL of each primer (5 μM), 0.4 μL of FastPfu Polymerase, and 10 ng of template DNA. The PCRs were conducted using the following program: 3 min of denaturation at 95°C; 27 cycles of 30 s at 95°C, 30 s for annealing at 55°C, and 45 s for elongation at 72°C; and a final extension at 72°C for 10 min. The quality of PCR products was tested using 2% agarose gel electrophoresis. Then, the resulting PCR products were extracted from a 2% agarose gel, further purified using an AxyPrep DNA Gel Extraction Kit (Axygen Biosciences, Union City, CA, United States), and quantified using QuantiFluorTM-ST (Promega, United States) according to the manufacturer’s protocol. The standard Illumina tags were added to PCR products using another PCR program. Then the tagged PCR products were extracted, purified, and checked. The single-stranded DNA was prepared. Purified amplicons were pooled in equimolar amounts and subjected to paired-end sequencing (2 × 300 bp) on an Illumina MiSeq platform (Illumina, San Diego, CA, United States) according to standard protocols. All samples were extracted and sequenced by Shanghai Majorbio Biopharm Technology Co., Ltd. (Shanghai, China).

The data for the current study could be obtained from the China National GeneBank DataBase (CNGBdb, https://db.cngb.org/) with the accession numbers CNP0002410 and CNP0002411, which are, respectively, continually updated aquatic wildlife gut microbiota data sets and continually updated aquatic environmental DNA data sets.

### Statistical analysis

The sequence data of each sample was analyzed on the Majorbio Cloud Platform (https://cloud.majorbio.com/). Raw fastq files were demultiplexed, quality-filtered by Trimmomatic (http://www.usadellab.org/cms/index.php?page=trimmomatic) and merged by FLASH (https://ccb.jhu.edu/software/FLASH/index.shtml). Operational taxonomic units (OTUs) were clustered with a 97% similarity cutoff using USEARCH7-uparse (http://drive5.com/uparse/), and chimeric sequences were identified and removed using UCHIME (http://www.drive5.com/uchime/uchime_download.html). The abundance of each OTU was analyzed using Usearch (http://www.drive5.com/usearch/). The taxonomy of each OTU was analyzed using the RDP Classifier Bayesian algorithm (http://sourceforge.net/projects/rdp-classifier/) against the database of silva138/16 s (http://www.arb-silva.de) with a confidence threshold of 75%. The phenotypes of each taxon were predicted using the phenotype prediction tool of BugBase (https://bugbase.cs.umn.edu/index.html), in which the unclassified sequences (sequences that cannot be annotated as species) were ignored in the analysis. The abundance ratios of microbes with the phenotypes of potential pathogenicity, mobile element content, and oxidative stress tolerance in each sample were obtained. Then, the microHI of each sample was calculated following Eq. 1. To identify the samples with disordered gut microbiota, hierarchical clustering (along with the column diagram of microbiota) of fish gut microbial samples was conducted at the microbial species, genus, and family levels based on the Bray–Curtis distance algorithm and the average linkage method (using Qiime http://qiime.org/install/index.html). The normal branches could be recognized using a definite species and even species with similar ecotypes, as we assumed that most individuals have a normal gut microbiota (Yang et al., 2022). The samples clustered in abnormal branches (out of normal branches) could be identified as disordered ones, which always indicates unhealthy hosts (Tong et al., 2020). The statistical analysis processes in the current study followed our previous work (Yang et al., 2023).

The current water environmental microbiota and fish gut microbiota data obtained from the Jiang’an section in June 2022 were analyzed to verify the relationship between gut microHI and disordered gut microbiota and then were combined with the data that was collected in 2020 at the sampling sections of Jiayu, Xinzhou, and Hukou in a previous study (Yang et al., 2023) to verify the correlation between gut microHI and environmental microHI at the fish community level. The data that was collected in 2020 includes 219 adult gut samples (29 from the Jiayu section, 156 from the Xinzhou section, and 34 from the Hukou section) from 11 species of fish and 43 water samples from two water environment groups (13 from the Xinzhou section, 30 from 30 sampling transects of the middle Yangtze River). Then, the gut microHI base value (average gut microHI of the individuals with normal gut microbiota) of each fish species was calculated. According to a pairwise comparison, the correlation relationship between gut microHI and disordered gut microbiota (the pairwise comparison of average gut microHIs from the samples with disordered gut microbiota and the samples with normal gut microbiota) and the correlation relationship between host growth condition (fullness) (Fulton’s condition, Eq. 2) (Bolger and Connolly, 1989; Hayes et al., 1995) and disordered gut microbiota (the pairwise comparison of average fullness between samples with disordered gut microbiota and samples with normal gut microbiota) were identified at the fish population level. A linear regression identified a correlation relationship between the gut microHI and environmental microHI at both the fish population level and the fish ecotype level. The gut microHI of each fish ecotype was adjusted using the fish community structure. In other words, the gut microHI of each fish ecotype was a weighted average of the gut microHI, where the individual percentage of each species in the fish community is the weighting factor. The key groups determining the microHI (with the phenotypes of potential pathogenicity, mobile element content, and oxidative stress tolerance) were identified using the non-parametric factorial Kruskal-Wallis (KW) sum-rank test and LDA (LDA ≥ 3).


(2) $$K=\frac{m}{L^{3}}\times 100$$

where *K* denotes Fulton’s condition (fullness) of an individual; *m* denotes the weight of an individual; and *L* denotes the body length of an individual (Bolger and Connolly, 1989; Hayes et al., 1995).

## Results

### The characteristics of the samples’ sequences

In the 214 adult fish gut samples from 14 species and 30 water environmental eDNA samples, we obtained 18,260,434 clean sequences, which were clustered into 15,247 OTUs and were identified as 1 kingdom, 65 phyla, 195 classes, 459 orders, 804 families, 1884 genera, and 2,409 species. The rarefaction curve (sobs & chaos, Supplementary Figures S1–S2) showed that the sequence depth was almost sufficient.

The abundance ratios of microbes with the phenotype of potential pathogenicity, mobile element content, or oxidative stress tolerance were variable in different samples and species (Supplementary Figure S3). More than half of the adult fish gut samples had a low (lower than 40%) relative abundance of the microbes with the phenotype of Potentially Pathogenic, a low (lower than 30%) relative abundance of the microbes with the phenotype of Contains Mobile Elements, and a low (lower than 20%) relative abundance of microbes with the phenotype of Stress Tolerant. Most of *Coilia brachygnathus* had a high (>50%) relative abundance of the microbes with the phenotypes of Potentially Pathogenic, Contains Mobile Elements, and Stress Tolerant. In 30 water environmental eDNA samples, the relative abundance of the microbes with the phenotype of Potentially Pathogenic mainly (>93%) ranges from 10 to 30%; the relative abundance of the microbes with the phenotype of Contains Mobile Elements mainly (90%) ranges from 50 to 70%; and the relative abundance of the microbes with the phenotype of Stress Tolerant mainly (>93%) is lower than 20%.

### The relationship among host health, gut microHI, and water environmental microHI at the community level

**Figure 1:**
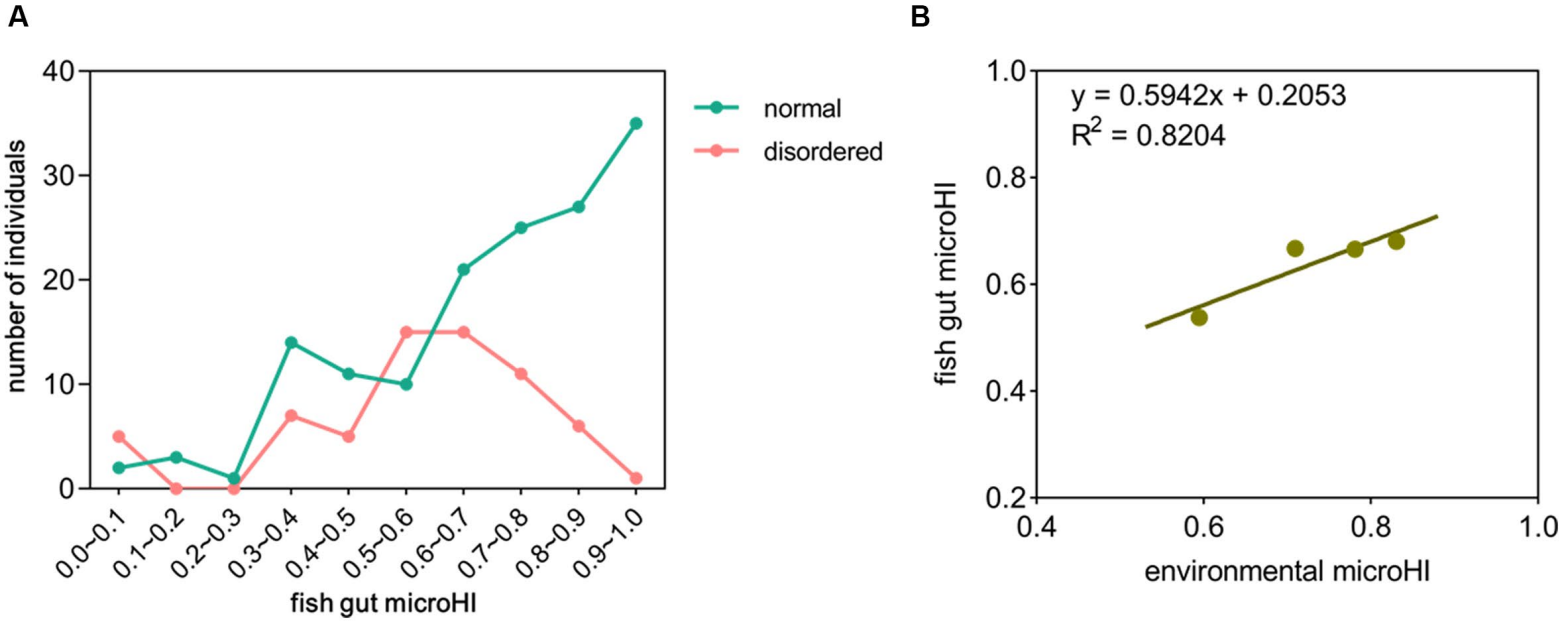
The correlation between fish gut microHI and water environmental microHI. **(A)** Distribution of fish gut microHIs in the Jiang’an section of the Yangtze River (*n* = 214). **(B)** The correlation between fish gut microHI (*n* = 370) and water environmental microHI (*n* = 43).

In the total of 214 fish gut microbial samples, 65 (30%) had disordered microbiota (unhealthy) (Supplementary Figures S4–S6). Approximately 66% of the fish gut microbial samples had a microHI of higher than 0.6, and approximately 15% of the fish gut microbial samples had a microHI of lower than 0.4. In the fish gut microbial samples with a microHI higher than 0.6, approximately 77% had normal microbiota (healthy). As the microHI increased, the probability of that the sample was captured from a healthy specimen also increased (Figure 1A). The average gut microHI of healthy individuals (0.71) was higher than that of unhealthy individuals (0.58). Comparing the microbial data from Jiang’an section in 2022 with the microbial data from Xinzhou section in 2020, the average environmental microHI of water samples is 0.71 (*n* = 30) in 2022, and 0.83 (*n* = 13) in 2020; the average gut microHI of all sampled individuals is 0.67 (*n* = 214) in 2022, and 0.68 (*n* = 156) in 2020; the average gut microHI of all sampled healthy fish individuals is 0.71 (*n* = 149) in 2022, and 0.73 (*n* = 115) in 2020; the percentage of healthy individuals was 69% in 2022 and 74% in 2020. Plotting the current diagram of the correlation between average fish gut microHI and average water environmental microHI with combined data (2020 and 2022), the positive correlation between the water environmental microHI and fish gut microHI at the community level was still maintained (Figure 1B).

### The relationship among host health, gut microHI, and water environmental microHI at the population level

**Figure 2:**
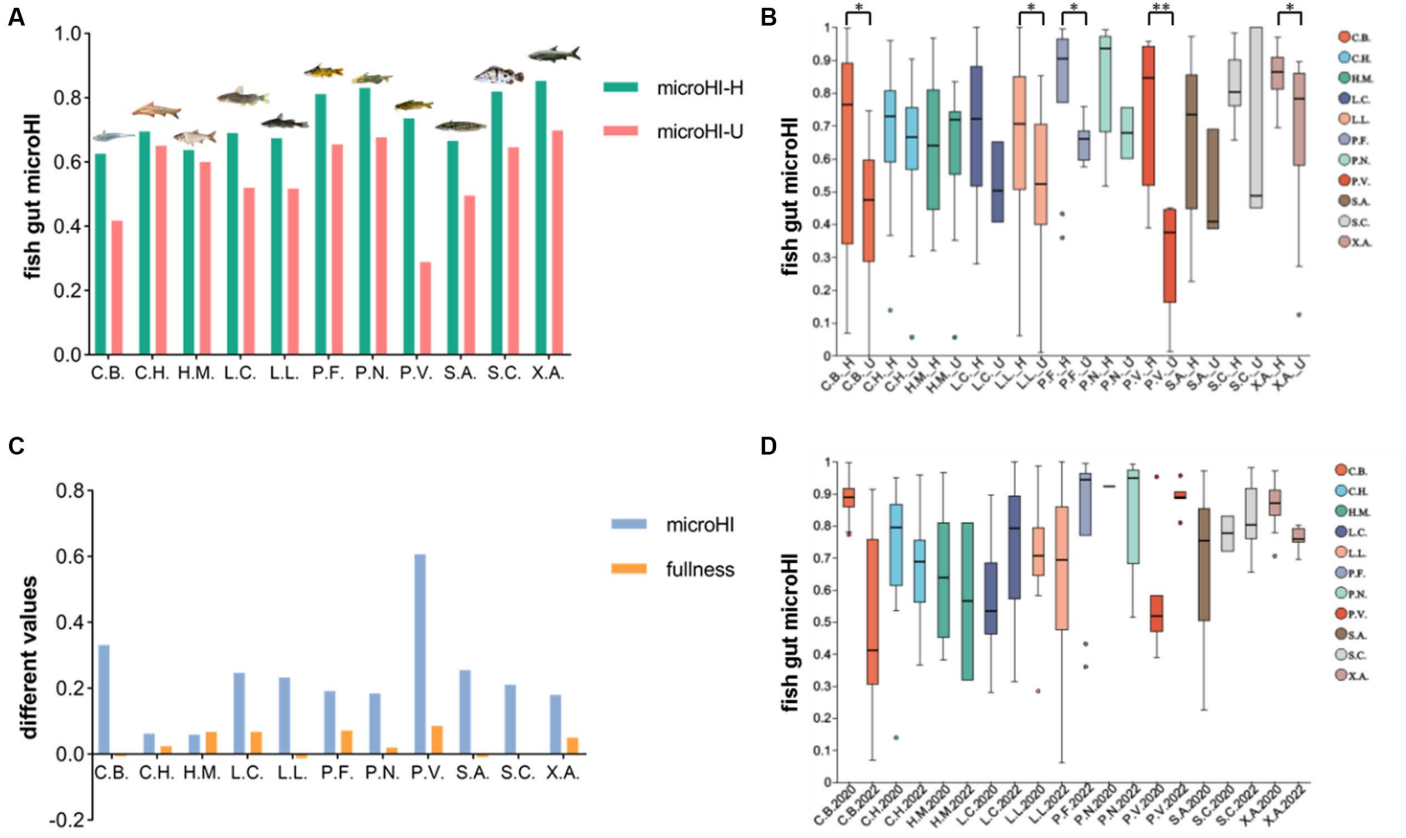
The gut microHIs of each species at the population level. **(A)** The average gut microHls of healthy (green) individuals and unhealthy (red) individuals for each fish species. **(B)** The gut microHl characters of healthy (_H) individuals and unhealthy (_U) individuals for each fish species. **(C)**, The difference in values of average microHl and average fullness (Fulton’s condition) between healthy and unhealthy groups of individuals. **(D)**, The gut microHls of healthy individuals for each fish species in 2020 and 2022. Eleven fish species groups: *C.B., Coilia brachygnathus; C.H., Coreius heterodon; H.M., Hypophthalmichthys molitrix L.C., Leiocassis crassilabris; (L).L., Leiocassis longirostris; (P).F., Pelteobagrus fulvidraco; P.N., Pelteobagrus nitidus; P.V., Pelteobagrus vachelli; (S).A., Silurus asotus; S.C., Siniperca chuatsi; (X).A., Xenocypris argentea*. *p < 0.05 and **p < 0.01 indicate significant differences.

In the combined data of the fish gut microbiota from the Jiang’an section in 2022 and the Xinzhou section in 2020, 11 fish species with more than 10 individuals were analyzed. The average gut microHI of healthy individuals was generally higher than that of unhealthy individuals in each fish species (Figure 2A). The gut microHI of the healthy group was significantly higher than that of the unhealthy group in some fish species (with enough samples in both healthy and unhealthy groups) (Figure 2B). The average Fulton’s condition of the healthy individuals was generally higher than the average Fulton’s condition of the unhealthy individuals in the majority of fish species, although the difference in Fulton’s condition was less than 9% (Figure 2C). As the water environmental microHI in 2020 was higher in 2020 than in 2022, the average gut microHI of the healthy fish group of a species was higher in 2020 than in 2022 for the majority of species (with three exceptions), although there was no significant difference between the sample groups in 2020 and 2022 (Figure 2D). The average gut microHI of healthy individuals in each species, which could be regarded as the base value of this species’ gut microHI, was species-specific (Figure 2A, Supplementary Table S2) and influenced by environmental microbiota (Figure 2D). Moreover, the degree to which the average value of the gut microHI in the healthy fish group of a species was higher in 2020 than in 2022 was found to be species-specific (Figure 2D). In other words, the influence of environmental microbiota on the base value of the gut microHI was species-specific, although there was a random error observed (Figure 2D).

### The relationship between gut microHI and water environmental microHI at the ecotype level

**Figure 3:**
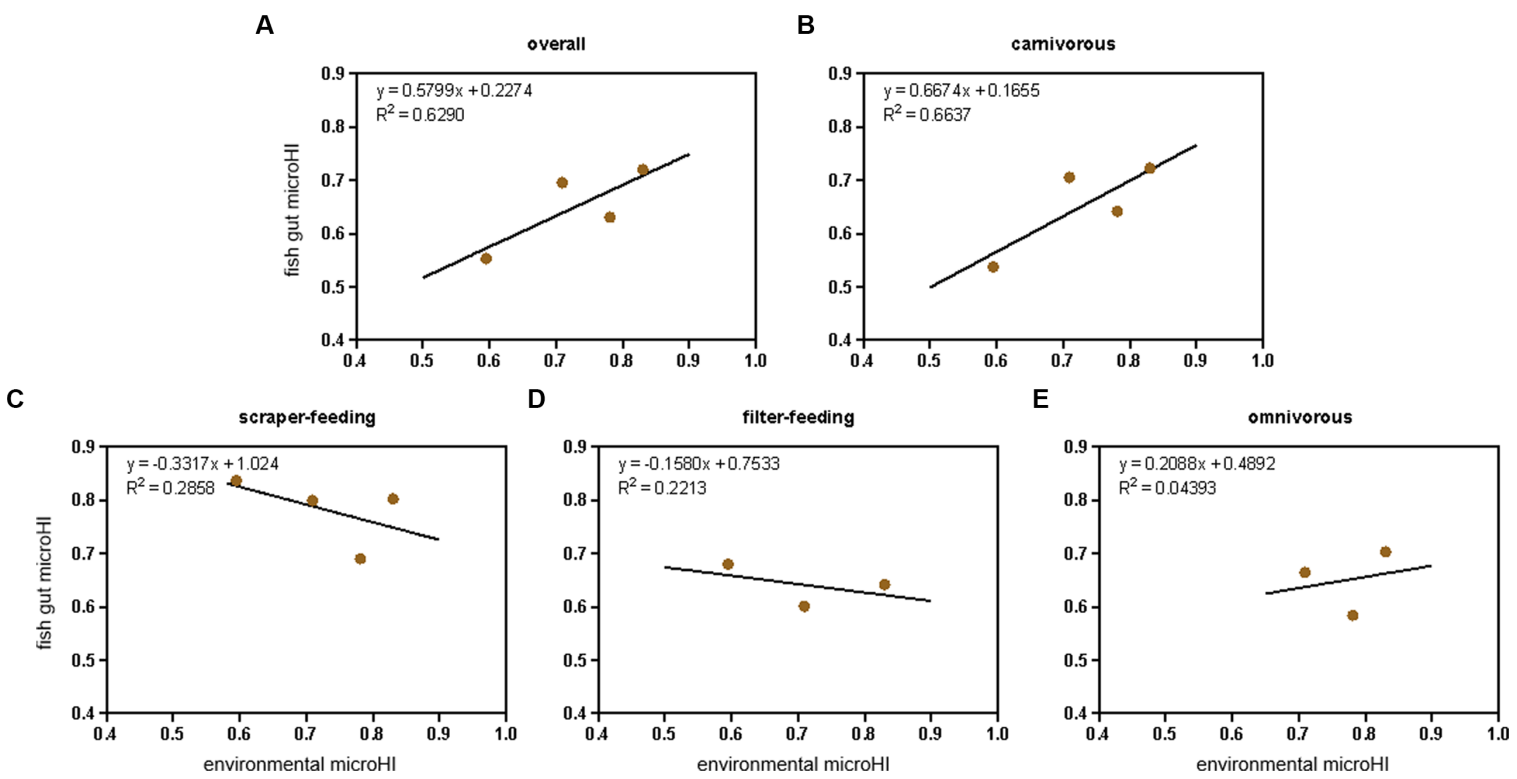
The adjusted correlation between water environmental microHI and fish gut microHI at the community level and ecotype level. The overall community level **(A)**, the ecotypes of carnivorous **(B)**, scraper-feeding **(C)**, filter-feeding **(D)**, and omnivorous **(E)** fish groups.

Considering the species-specific relationship between gut microHI and environmental microHI, the community-level fish gut microHI of each sampling location (Jiayu, Jiang’an, Xinzhou, and Hukou) was corrected using the fish community structure (based on the number of individuals, Supplementary Table S3) identified by our conventional Yangtze fishery resource investigation. The fish in the community were divided into four ecotypes: filter-feeding, scraper-feeding, omnivorous, and carnivorous (Table 1). The positive correlation between water environmental microHI and fish gut microHI at the community level was still maintained (Figure 3A), while the positive correlation at the ecotype level was only maintained in the carnivorous fish group (Figures 3B-E).

### The key groups that potentially impact the health status of wild fish from water environmental microbiota

When analyzing the fish gut microbiota and the water environmental microbiota, there were mainly 107 families and 106 genera that related to the phenotypes of potential pathogenicity, mobile element content, and oxidative stress tolerance (Supplementary Figures S7–S12). The most abundant groups with a negative impact on fish gut microHI were Alcaligenaceae, Clostridiaceae, Peptostreptococcaceae, Enterobacteriaceae, and Moraxellaceae at the family level, and *Achromobacter*, *Plesiomonas*, *Clostridium*, *Aeromonas*, and *Acinetobacter* at the genus level (Supplementary Figures S7–S12). A total of 26 families and 22 genera had high correlations (r^2^ > 0.6) between the gut sample group and environment sample group, among which 17 families and 13 genera had positive correlations (slope > 0.1), including Alcaligenaceae, Enterobacteriaceae, and *Achromobacter* (Supplementary Table S4).

**Figure 4:**
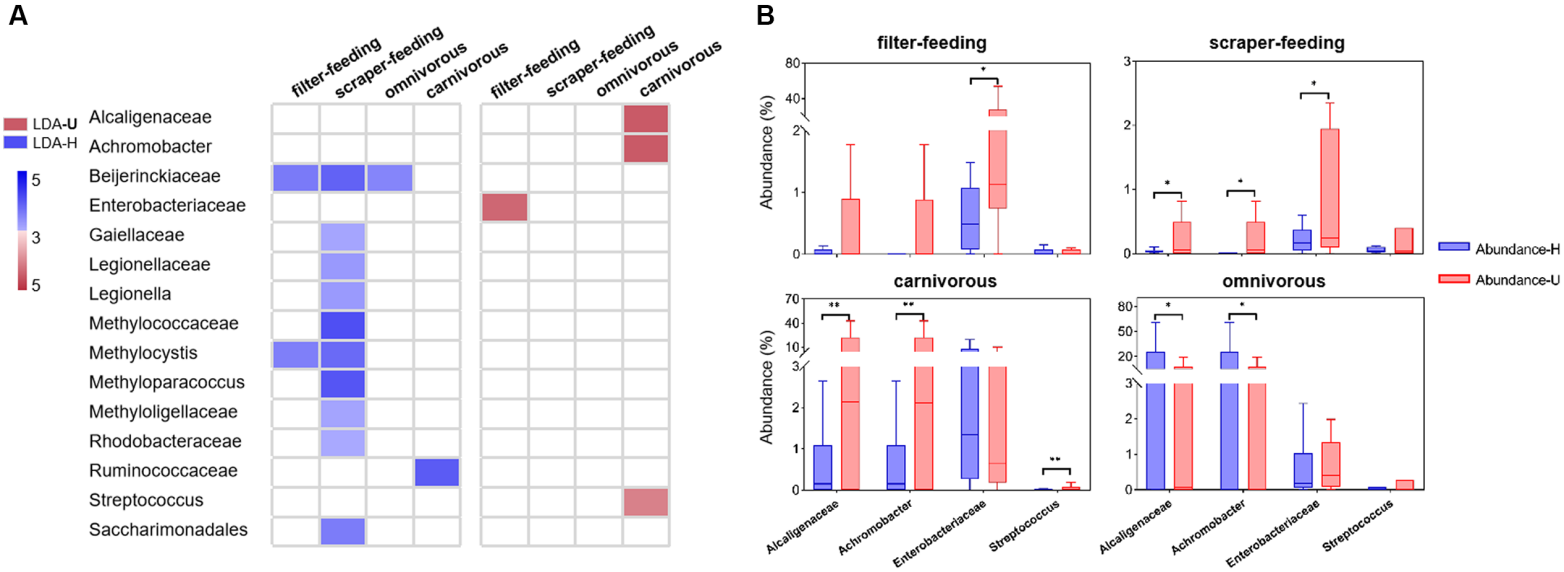
Differentially abundant bacterial taxa from the healthy and unhealthy groups in four ecotypes. **(A)**, A plot showing taxa that were significantly differentially abundant between the healthy (blue) and unhealthy (red) groups, as determined using the Kruskal-Wallis test. The LDA score (effect size) indicates significant differences in bacterial taxa (LDA score > 3.0; alpha value *p* < 0.05). **(B)**, Relative abundance of indicator taxa in the healthy (blue) and unhealthy (red) groups. *p < 0.05 and **p < 0.01 indicate significant differences.

Comparing the healthy groups and unhealthy groups of the four ecotypes (filter-feeding, scraper-feeding, omnivorous, and carnivorous), the percentage of families and genera that related to the phenotypes of potential pathogenicity, mobile element content, and oxidative stress tolerance was lower in the healthy group than in the unhealthy group (Supplementary Table S5). There were 51 families and 47 genera that had significant abundance differences between healthy and unhealthy groups (Supplementary Figures S13–S16 and Supplementary Table S5), among which 10 families and 6 genera showed positive correlations (slope > 0.1, r^2^ > 0.6) between gut sample groups and environment sample groups in each ecotype (except the omnivorous ecotype) (Figure 4A and, Supplementary Tables S4–S5). Additionally, only two families (Alcaligenaceae, Enterobacteriaceae) and two genera (*Achromobacter*, *Streptococcus*) had significantly higher abundance in the unhealthy groups than in the healthy groups of the carnivorous, filter-feeding, and scraper-feeding ecotypes (Figure 4B). The other families and genera did not have significantly higher abundances in unhealthy groups than in healthy groups.

## Discussion

### Gut microHI could indicate the health status of wild fish at both community and population levels

The homeostasis of the intestinal system occupies an important position in the regulation of the host immune system, health, and physiology (Su et al., 2018; Zhong et al., 2019). The metabolites of gut microbes can act as energy sources for cell metabolism, as promoters of development and immune function, and can prevent colonization by pathogenic microorganisms (Honda and Littman, 2012; Butt and Volkoff, 2019). The altered gut microbiota composition can affect fish health and is often associated with a diseased state (Sartor and Wu, 2017; Sun et al., 2020; Bozzi et al., 2021). Many studies have shown that the gut microbiota can be a monitoring indicator of multiple diseases (Xiong et al., 2019; Wang et al., 2022), abnormal physiological metabolism (Kraimi et al., 2019; Kim et al., 2020), and general health conditions (Borbón-García et al., 2017; Bozzi et al., 2021) in mammals, birds, fish, etc.

The gut microHI could reflect the microbial composition associated with tissue damage, risk of disease, or potential threats to host health (Sáenz et al., 2019; Himani et al., 2020). One of our previous studies found that, at the community level, most of the fish gut samples with low microHI were obtained from specimens with a disordered gut microbiota, which is always indicative of an unhealthy host, so the gut microHI could potentially be used to evaluate host health status (Yang et al., 2023). In the current study, the new data showed that, at the community level, (1) the individuals with normal gut microbiota were more likely to have a higher gut microHI than the individuals with disordered gut microbiota, and (2) the individuals with a higher gut microHI were more likely to have normal gut microbiota than the individuals with a lower gut microHI (Figure 1A). In addition, the average gut microHI of healthy individuals was higher than that of unhealthy individuals. Thus, the current data support the idea that the gut microHI could be used to indicate host health status at the community level.

In the 11 fish species analyzed, the average Fulton’s condition of healthy individuals was found to be generally higher than that of unhealthy individuals in the majority of fish species, although the difference in Fulton’s condition was small (Figure 2C). Generally speaking, in a given species, a higher Fulton’s condition in an adult fish individual always indicates a more healthy status (Nash et al., 2006), although the Fulton’s condition index has some limitations (Bolger and Connolly, 1989; Hayes et al., 1995). Therefore, the delineation of a healthy group based on normal gut microbiota is supported by the growth status of individuals as indicated by Fulton’s condition. Moreover, the average gut microHI of healthy individuals was generally higher than that of unhealthy individuals in each species, although there was individual variation (Figures 2A–B). This suggests that the gut microHI can be used to indicate host health status at the population level.

### The correlation between gut microHI and water environmental microHI is positive at the community level, but variable at the population and ecotype levels

Environmental microbiota (Zhang et al., 2021; Zhou et al., 2021), diet composition (Gajardo et al., 2017; Wilkes Walburn et al., 2019), host genetics (Zhang et al., 2019; Callens et al., 2020), and environmental pollutants (Li et al., 2022; Zhang et al., 2023) have been demonstrated to play important roles in defining the composition of the gut microbiota of the host. In aquatic animals, the introduction of environmental microbes is known to be an extremely important force in constructing the gut microbiota (Zhang et al., 2021; Zhou et al., 2021; Yang et al., 2022). One of our previous studies found that there were low fish gut microHIs in the Yangtze River sections with low aquatic environmental microHI (Yang et al., 2023), which is consistent with the current results of a positive correlation between the gut microHI and the water environmental microHI at the community level. The current results support the idea that the water environmental microHI could be used to indicate the gut microHI at the community level. Moreover, the adjusted average fish gut microHI of the sampling locations still maintained a positive correlation between the water environmental microHI and fish gut microHI (Figure 3A). Therefore, considering that the gut microHI could indicate the health status of wild fish and that there is a positive correlation between the water environmental microHI and the fish gut microHI at the community level, the water environmental microHI could serve as a useful indicator of the health status of the wild fish community unit.

The base values of the gut microHI for each species, indicated by the mean gut microHI of healthy individuals, were species-specific at the population level (Figure 2A and Supplementary Table S2), and the water environmental effects were similarly species-specific (Figure 2D). These species variations are impacted by the combined effects of host-genetic deterministic selection and the introduction of neutral microbes (Ley et al., 2011; Ringø et al., 2016; Legrand et al., 2020). The host-genetic deterministic selection depends on the host digestive system structures (Yan et al., 2016; Wilkes Walburn et al., 2019; Xiao et al., 2021). The introduction of neutral microbes depends on the habitats and diet conditions of the hosts (Ringø et al., 2016; Kashinskaya et al., 2018; Sun et al., 2019; Heys et al., 2020). Of course, part of the variations in the current work may have been caused by individual random errors. The correlation between fish gut microHI and water environmental microHI is variable at the population level.

Host-mediated environmental factors such as diet, lifestyle, xenobiotic exposure, and medications can induce substantial shifts in microbiome composition (Yatsunenko et al., 2012; Schroeder et al., 2016). Multiple studies have indicated associations between long-term diet and host microbiota (Turnbaugh et al., 2009; Albenberg and Wu, 2014; Cresci and Bawden, 2015). Diet was also the main force influencing gut microbial diversity (Falony et al., 2016; Turpin et al., 2016; Zhernakova et al., 2016). However, it only explained a small percentage of the variation in the microbiota. Thus, given the importance of diet on the gut microbiota composition, we combined the populations into four ecotypes (filter-feeding, scraper-feeding, omnivorous, and carnivorous, Table 1). The results showed that only the carnivorous group maintained a positive correlation between gut microHI and water environmental microHI (Figures 3B-E), whereas the filter-feeding, scraper-feeding, and omnivorous groups did not. Perhaps driven by the extensive and longer intestinal system (filter-feeding, 5.0–6.0 times body length; scraper-feeding, 1.5–5.0 times body length; omnivorous, 0.9–1.1 times body length) (Chen, 1998), there was a relatively stable gut microbiota structure in filter-feeding, scraper-feeding, and omnivorous species that was seldom or weakly influenced by environmental microbiota (Figures 3C-E). In contrast, the short (0.3–0.7 times body length) intestinal system of carnivorous fish (Zheng, 1987; Zhu et al., 1999) was strongly influenced by environmental microbiota; therefore, there was a positive correlation between gut microHI and water environmental microHI (Figure 3B). Of course, it is possible that the effects of sediment environmental microbiota on the gut microbiota structure of the scraper-feeding and omnivorous groups (Zhang et al., 2021; Zhou et al., 2021; Yang et al., 2022) caused the weak correlation between the gut microHI of the scraper-feeding and omnivorous species and water environmental microHI.

### Alcaligenaceae, Enterobacteriaceae (family level), and Achromobacter (genus level) are the key groups that may impact the health status of wild fish from environmental microbiota in water

The groups that have a negative influence on the health status of wild fish are general in both the gut microbiota and the environmental microbiota (Al-Hisnawi et al., 2016). The aquatic habitat, as a living matrix, supports various microbes (Legrand et al., 2020; Sehnal et al., 2021). The gut of aquatic wild animals, due to sharing a large amount of microbes with the environment (Yang et al., 2022), also harbors various microbes. In the current results, there were mainly 107 families and 106 genera that related to the phenotypes of potential pathogenicity, mobile element content, and oxidative stress tolerance, and a large amount of these groups were present in most samples (including from both healthy and unhealthy individuals) (Supplementary Figures S7–S12).

Few of the groups showed the possibility of originating from water environmental microbiota and potentially impacting the health status of wild fish. In the groups that related to the phenotypes of potential pathogenicity, mobile element content, and oxidative stress tolerance, the abundance of 26 families and 22 genera in gut sample group was strongly correlated with that in environment sample group (r^2^ > 0.6), and the abundance correlations were positive for 17 families and 13 genera (slope > 0.1) (Supplementary Table S4). Comparing the healthy groups and unhealthy groups of four ecotypes, 10 families and 6 genera (part of previous 17 families and 13 genera) had significant abundance difference between healthy groups and unhealthy groups (Supplementary Figures S13–S16 and Supplementary Table S5), and only two families (Alcaligenaceae, Enterobacteriaceae) and two genera (*Achromobacter*, *Streptococcus*) had significantly high abundance in the unhealthy group of the carnivorous or filter-feeding ecotypes. Furthermore, Alcaligenaceae, Enterobacteriaceae (family level), and *Achromobacter* (genus level) were found to be the most abundant groups among the 107 families and 106 genera. Alcaligenaceae, including the genus *Achromobacter*, as opportunistic pathogens, are significantly more abundant in hosts with certain diseases (Swenson and Sadikot, 2015; Pan et al., 2020), and can be a bacterial bio-indicator of environmental pollution (Zolkefli et al., 2020). Enterobacteriaceae are an important normal flora in the intestine. However, some species can become opportunistic pathogens and cause infections after the host’s immunity declines, resulting in a variety of diseases (Satlin and Walsh, 2017; Skuja et al., 2018; Pisani et al., 2021). In the current results, Alcaligenaceae, including the genus *Achromobacter*, are associated with potential pathogenicity and oxidative stress tolerance. Enterobacteriaceae are related to potential pathogenicity, mobile elements, and oxidative stress tolerance. Therefore, we believe that Alcaligenaceae, Enterobacteriaceae (family level), and *Achromobacter* (genus level) are the key water environmental microbial groups that potentially impact the health status of wild fish in the middle Yangtze River.

### A weighted microHI, based on scientific rationale, should be a better indicator of the health status of wild fish

In one of our previous works, microHI was defined as the unweighted average abundance ratio of microbes without three health-negative-impacting microbial phenotypes, i.e., potential pathogenicity, mobile element content, and oxidative stress tolerance (Eq. 1) (Yang et al., 2023). MicroHI has shown its ability to evaluate the health status of wild fish and the possibility of using water environmental microbiota to indicate the health status of wild fish (Yang et al., 2023). However, the current unweighted microHI is only a primary indicator (microHI-1.0). The correlation between fish gut microHI and the delineation of healthy and unhealthy individuals in a species is not very accurate (little exceptions) and strong (high r2) (Figure 1A). The correlation between fish gut microHI and environmental microHI is also not very accurate (little exceptions) and strong (high r2) (Figure 3). Perhaps a weighted microHI based on scientific evidence would be the best way forward.

Although Potentially Pathogenic, Contains Mobile Elements, and Stress Tolerant are identified as health-negative-impacting microbial phenotypes, in practice, the microorganism with these phenotypes could be beneficial if it had low abundance and low health-negative-impacting (Wiles and Guillemin, 2019). Using the microHI-1.0, this situation cannot be taken into account when assessing the health status of fish using the fish gut microHI. Then, the effectiveness of using water environmental microHI to predict the health status of wild fish could also be impacted. Therefore, an updated microHI (microHI-2.0) that considers these influences might be better. To obtain the microHI-2.0, sufficient scientific data and knowledge are needed to determine the weighting factors and adjustment parameters. More studies in this field are necessary. We hope that microHI-2.0 can be realized in the future.

## Conclusion

The current work showed that the gut microHI could indicate the community health status of wild fish and had a positive correlation with water environmental microHI. In other words, the current results support the idea that the water environmental microHI could be used to indicate the health status of wild fish at the community level. Moreover, the current study indicated that the gut microHI could indicate the population health status of wild fish, although it showed a variable correlation with water environmental microHI. When fish species were grouped into four ecotypes, only carnivorous fish retained the positive correlation between fish gut microHI and water environmental microHI. Furthermore, Alcaligenaceae, Enterobacteriaceae (family level), and *Achromobacter* (genus level) were the key groups introduced from the water environmental microbiota and potentially impacted the health status of wild fish in the middle Yangtze River. Therefore, this study comprehensively verifies the effectiveness of water environmental microHI as an indicator of the health status of wild fish. Our work provides a non-invasive method for the health monitoring of wild fish, offers a new path for understanding and managing aquatic wild animals’ health status, and provides a new tool for the aquatic wildlife conservation toolkit. Of course, more research to develop the microHI-2.0 with weighting factors and adjustment parameters is welcome.

## Data availability statement

The datasets presented in this study can be found in online repositories. The names of the repository/repositories and accession number(s) can be found in the article/Supplementary material.

## Author contributions

HY: Conceptualization, Data curation, Formal analysis, Funding acquisition, Investigation, Methodology, Software, Visualization, Writing – original draft, Writing – review & editing. JZ: Data curation, Formal analysis, Visualization, Writing – original draft, Writing – review & editing. XL: Writing – review & editing, Methodology. JMW: Resources, Writing – review & editing. PC: Writing – review & editing. LS: Project administration, Writing – review & editing. JPW: Writing – review & editing. PL: Writing – review & editing. HD: Funding acquisition, Supervision, Validation, Writing – review & editing.
