# Supplementary material for: Effectiveness assessment of using water environmental microHI to predict the health status of wild fish
Source: Front Microbiol. 2024 Jan 11;14:1293342. doi: 10.3389/fmicb.2023.1293342 (PMC10808811; doi:10.3389/fmicb.2023.1293342)
Supplement: Supplementary file 1 [file Presentation_1.PPTX]

## Slide 1
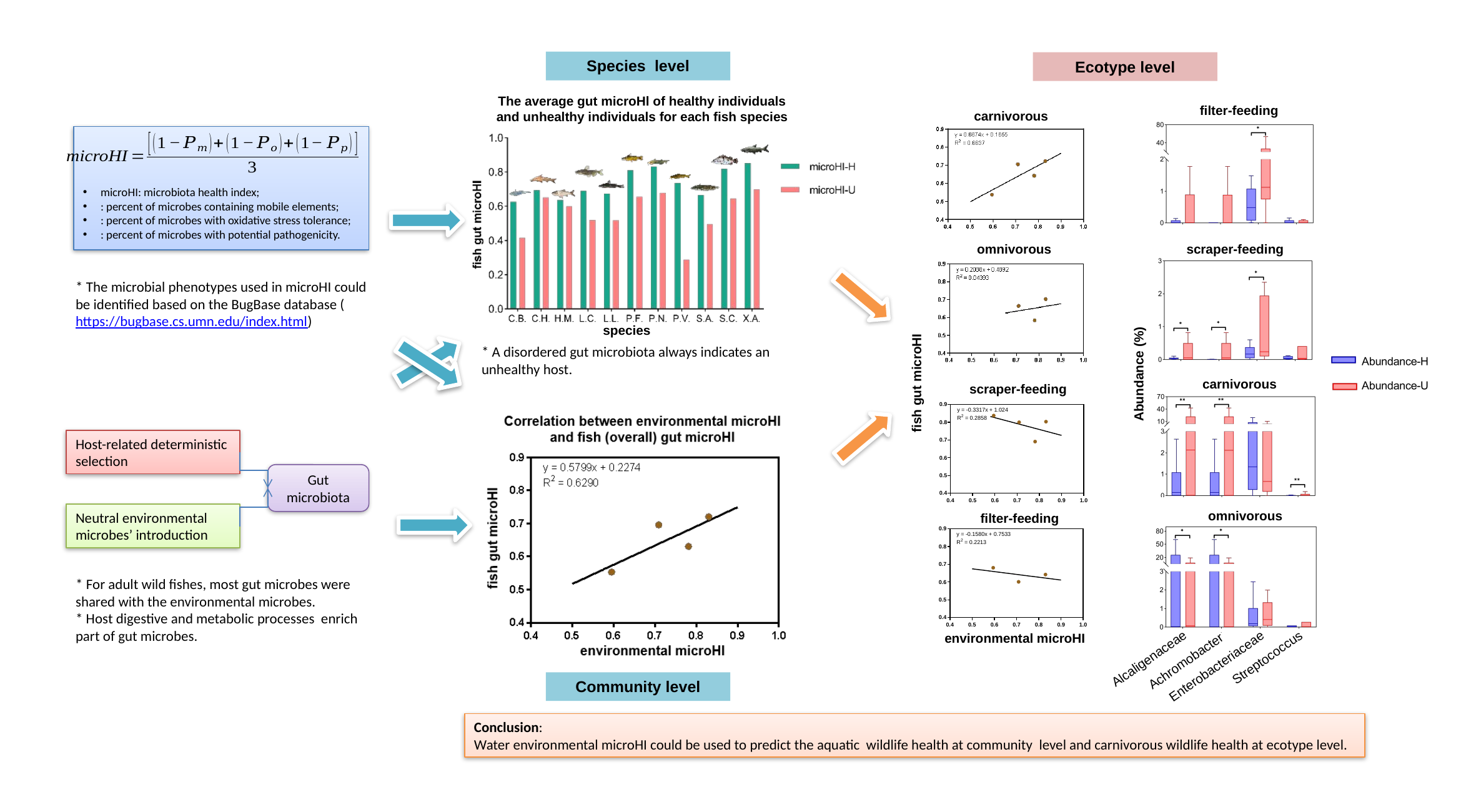

Species level
Ecotype level
The average gut microHl of healthy individuals and unhealthy individuals for each fish species
filter-feeding
carnivorous
* The microbial phenotypes used in microHI could be identified based on the BugBase database (https://bugbase.cs.umn.edu/index.html)
omnivorous
scraper-feeding
species
* A disordered gut microbiota always indicates an unhealthy host.
Abundance (%)
fish gut microHI
carnivorous
scraper-feeding
Host-related deterministic selection
Gut microbiota
Neutral environmental microbes’ introduction
* For adult wild fishes, most gut microbes were shared with the environmental microbes.
* Host digestive and metabolic processes enrich part of gut microbes.
omnivorous
filter-feeding
environmental microHI
Streptococcus
Alcaligenaceae
Achromobacter
Enterobacteriaceae
Community level
Conclusion:
Water environmental microHI could be used to predict the aquatic wildlife health at community level and carnivorous wildlife health at ecotype level.
