# Supplementary material for: Effectiveness assessment of using water environmental microHI to predict the health status of wild fish
Source: Front Microbiol. 2024 Jan 11;14:1293342. doi: 10.3389/fmicb.2023.1293342 (PMC10808811; doi:10.3389/fmicb.2023.1293342)
Supplement: Supplementary file 2 [file Data_Sheet_1.ZIP › Supplementary Figure S13 LDA filter-feeding.pdf]

Cladogram

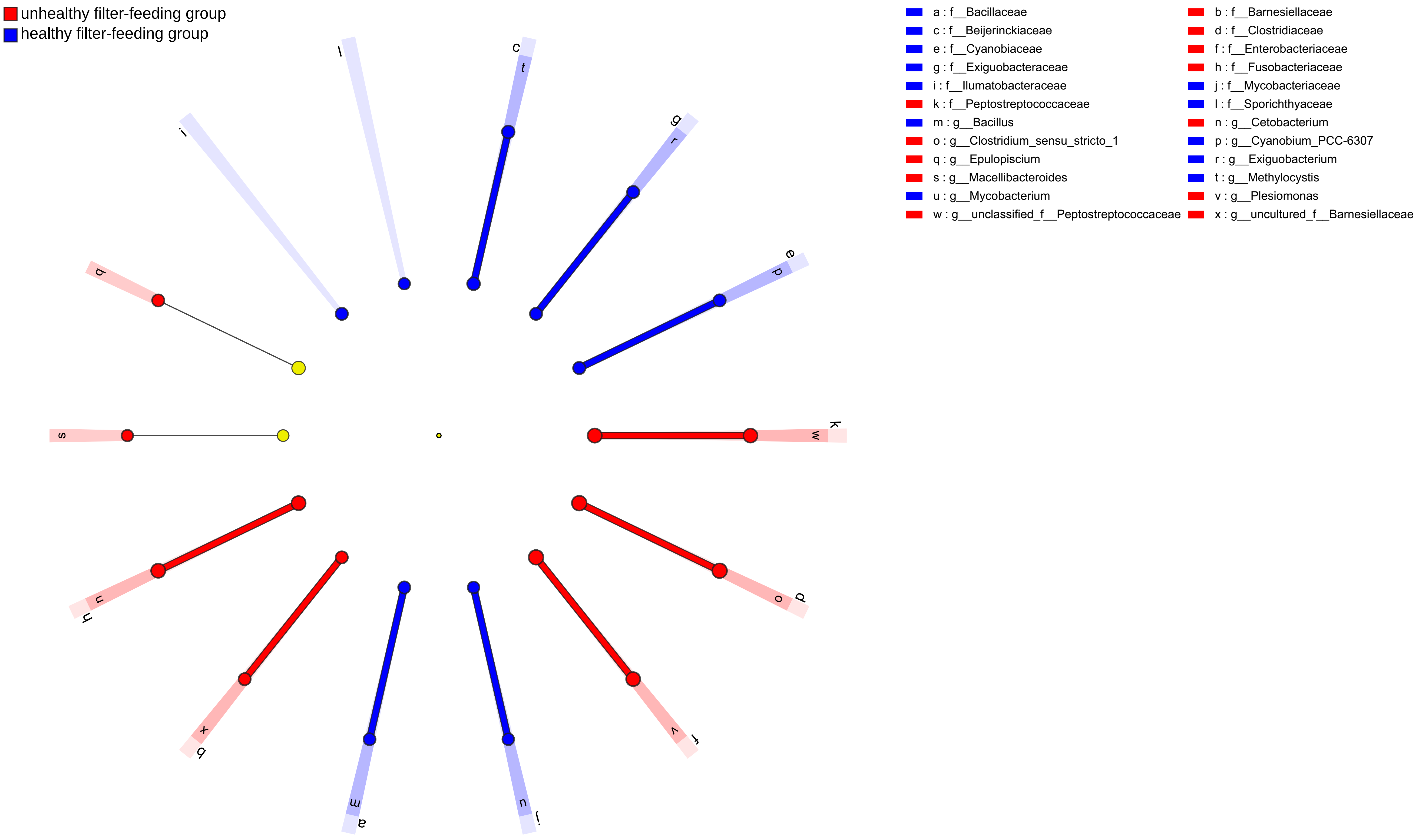

unhealthy filter-feeding group
healthy filter-feeding group
a : f__Bacillaceae
b : f__Barnesiellaceae
c : f__Beijerinckiaceae
d : f__Clostridiaceae
e : f__Cyanobiaceae
f : f__Enterobacteriaceae
g : f__Exiguobacteraceae
h : f__Fusobacteriaceae
i : f__Ilumatobacteraceae
j : f__Mycobacteriaceae
k : f__Peptostreptococcaceae
l : f__Sporichthyaceae
m : g__Bacillus
n : g__Cetobacterium
o : g__Clostridium_sensu_stricto_1
p : g__Cyanobium_PCC-6307
q : g__Epulopiscium
r : g__Exiguobacterium
s : g__Macellibacteroides
t : g__Methylocystis
u : g__Mycobacterium
v : g__Plesiomonas
w : g__unclassified_f__Peptostreptococcaceae
x : g__uncultured_f__Barnesiellaceae
