# Supplementary material for: Effectiveness assessment of using water environmental microHI to predict the health status of wild fish
Source: Front Microbiol. 2024 Jan 11;14:1293342. doi: 10.3389/fmicb.2023.1293342 (PMC10808811; doi:10.3389/fmicb.2023.1293342)
Supplement: Supplementary file 2 [file Data_Sheet_1.ZIP › Supplementary Figure S14 LDA scraper-feeding.pdf]

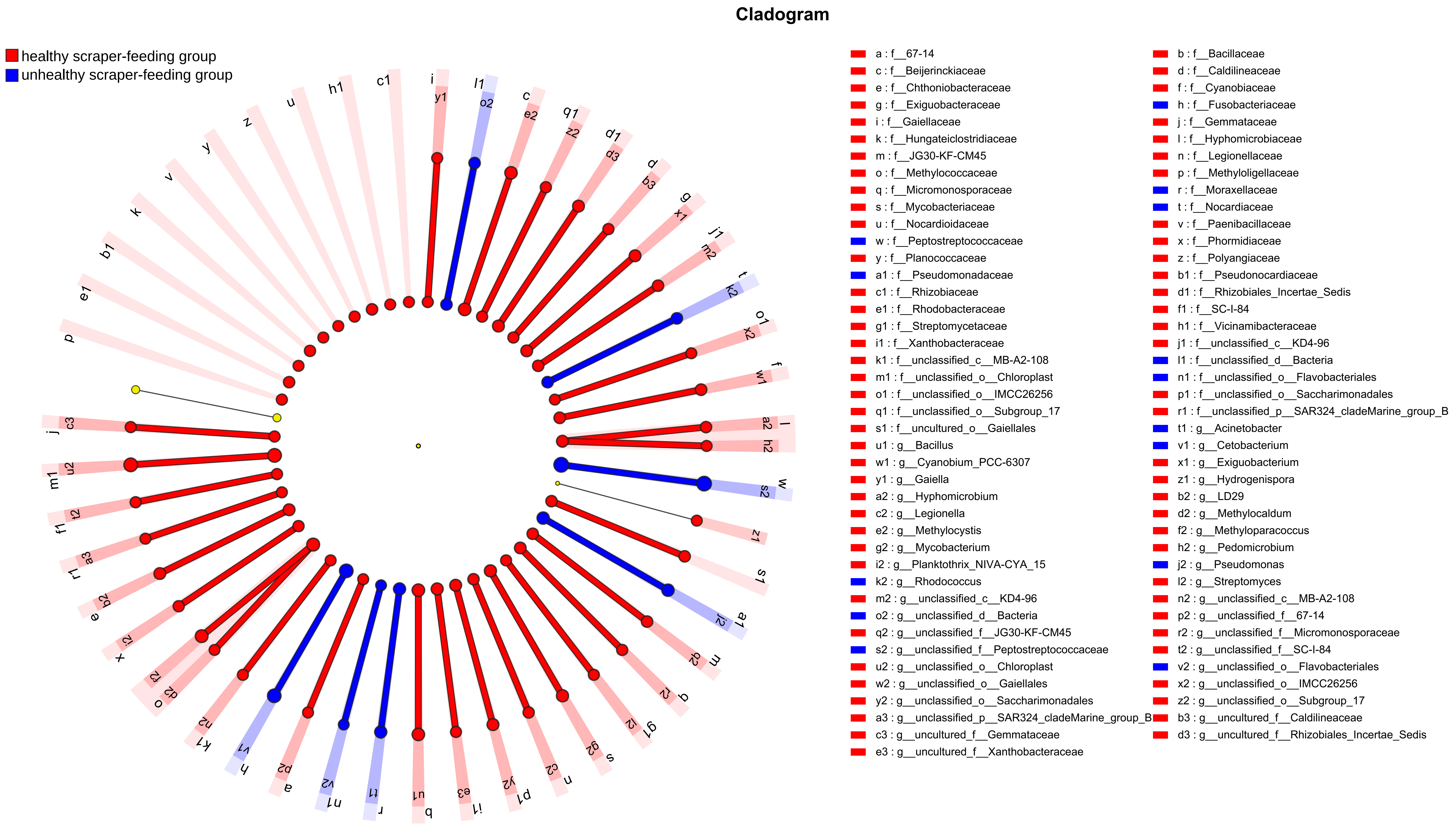
Cladogram
healthy scraper-feeding group
unhealthy scraper-feeding group
a : f__67-14
c : f__Beijerinckiaceae
e : f__Chthoniobacteraceae
g : f__Exiguobacteraceae
i : f__Gaiellaceae
k : f__Hungateiclostridiaceae
m : f__JG30-KF-CM45
o : f__Methylococcaceae
q : f__Micromonosporaceae
s : f__Mycobacteriaceae
u : f__Nocardioidaceae
w : f__Peptostreptococcaceae
y : f__Planococcaceae
a1 : f__Pseudomonadaceae
c1 : f__Rhizobiaceae
e1 : f__Rhodobacteraceae
g1 : f__Streptomycetaceae
i1 : f__Xanthobacteraceae
k1 : f__unclassified_c__MB-A2-108
m1 : f__unclassified_o__Chloroplast
o1 : f__unclassified_o__IMCC26256
q1 : f__unclassified_o__Subgroup_17
s1 : f__uncultured_o__Gaiellales
u1 : g__Bacillus
w1 : g__Cyanobium_PCC-6307
y1 : g__Gaiella
a2 : g__Hyphomicrobium
c2 : g__Legionella
e2 : g__Methylocystis
g2 : g__Mycobacterium
i2 : g__Planktothrix_NIVA-CYA_15
k2 : g__Rhodococcus
m2 : g__unclassified_c__KD4-96
o2 : g__unclassified_d__Bacteria
q2 : g__unclassified_f__JG30-KF-CM45
s2 : g__unclassified_f__Peptostreptococcaceae
u2 : g__unclassified_o__Chloroplast
w2 : g__unclassified_o__Gaiellales
y2 : g__unclassified_o__Saccharimonadales
a3 : g__unclassified_p__SAR324_cladeMarine_group_B
c3 : g__uncultured_f__Gemmataceae
e3 : g__uncultured_f__Xanthobacteraceae
b : f__Bacillaceae
d : f__Caldilineaceae
f : f__Cyanobiaceae
h : f__Fusobacteriaceae
j : f__Gemmataceae
l : f__Hyphomicrobiaceae
n : f__Legionellaceae
p : f__Methyloligellaceae
r : f__Moraxellaceae
t : f__Nocardiaceae
v : f__Paenibacillaceae
x : f__Phormidiaceae
z : f__Polyangiaceae
b1 : f__Pseudonocardiaceae
d1 : f__Rhizobiales_Incertae_Sedis
f1 : f__SC-I-84
h1 : f__Vicinamibacteraceae
j1 : f__unclassified_c__KD4-96
l1 : f__unclassified_d__Bacteria
n1 : f__unclassified_o__Flavobacteriales
p1 : f__unclassified_o__Saccharimonadales
r1 : f__unclassified_p__SAR324_cladeMarine_group_B
t1 : g__Acinetobacter
v1 : g__Cetobacterium
x1 : g__Exiguobacterium
z1 : g__Hydrogenispora
b2 : g__LD29
d2 : g__Methylocaldum
f2 : g__Methyloparacoccus
h2 : g__Pedomicrobium
j2 : g__Pseudomonas
l2 : g__Streptomyces
n2 : g__unclassified_c__MB-A2-108
p2 : g__unclassified_f__67-14
r2 : g__unclassified_f__Micromonosporaceae
t2 : g__unclassified_f__SC-I-84
v2 : g__unclassified_o__Flavobacteriales
x2 : g__unclassified_o__IMCC26256
z2 : g__unclassified_o__Subgroup_17
b3 : g__uncultured_f__Caldilineaceae
d3 : g__uncultured_f__Rhizobiales_Incertae_Sedis
