# Supplementary material for: Effectiveness assessment of using water environmental microHI to predict the health status of wild fish
Source: Front Microbiol. 2024 Jan 11;14:1293342. doi: 10.3389/fmicb.2023.1293342 (PMC10808811; doi:10.3389/fmicb.2023.1293342)
Supplement: Supplementary file 2 [file Data_Sheet_1.ZIP › Supplementary Figure S3 Phenotypes.pptx]

## Slide 1
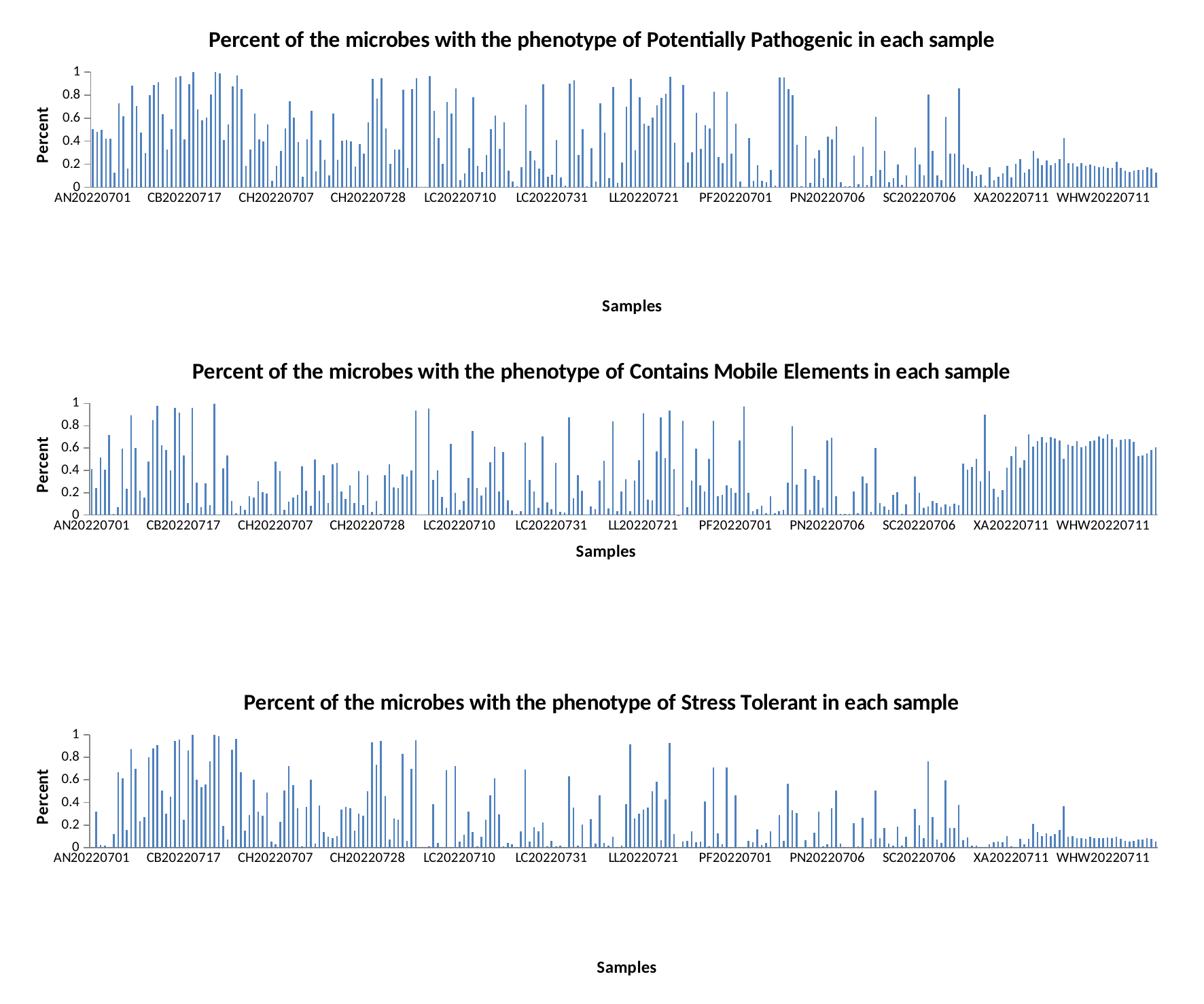

### Chart: Percent of the microbes with the phenotype of Potentially Pathogenic in each sample
| Category | Potentially_Pathogenic |
|---|---|
| AN20220701 | 0.504566969075 |
| AN20220702 | 0.479754443846 |
| AN20220703 | 0.499593263639 |
| AN20220704 | 0.422152581389 |
| AN20220705 | 0.420024349238 |
| CB20220701 | 0.124632676893 |
| CB20220702 | 0.726656072939 |
| CB20220703 | 0.615091065973 |
| CB20220704 | 0.161450851324 |
| CB20220705 | 0.879512078261 |
| CB20220706 | 0.705102797615 |
| CB20220707 | 0.472346099333 |
| CB20220708 | 0.298233833616 |
| CB20220709 | 0.801762114537 |
| CB20220710 | 0.884712345261 |
| CB20220711 | 0.908319466369 |
| CB20220712 | 0.633469954045 |
| CB20220713 | 0.329313934152 |
| CB20220714 | 0.50289527685 |
| CB20220715 | 0.953338481631 |
| CB20220716 | 0.962791581372 |
| CB20220717 | 0.415095858754 |
| CB20220718 | 0.892147084229 |
| CB20220719 | 0.996858212284 |
| CB20220720 | 0.678295818563 |
| CB20220721 | 0.578930984333 |
| CB20220722 | 0.607179944352 |
| CB20220723 | 0.803294140503 |
| CB20220724 | 1.0 |
| CB20220725 | 0.986088644452 |
| CB20220726 | 0.412081485355 |
| CB20220727 | 0.547279462396 |
| CB20220728 | 0.874204634838 |
| CB20220729 | 0.971123866364 |
| CB20220730 | 0.853511383759 |
| CB20220731 | 0.188028895817 |
| CH20220701 | 0.328282337371 |
| CH20220702 | 0.638591316468 |
| CH20220703 | 0.415379344138 |
| CH20220704 | 0.395893532355 |
| CH20220705 | 0.545140080801 |
| CH20220706 | 0.0572273838072 |
| CH20220707 | 0.183332585873 |
| CH20220708 | 0.313755511181 |
| CH20220709 | 0.51331288495 |
| CH20220710 | 0.746147611561 |
| CH20220711 | 0.606469519127 |
| CH20220712 | 0.394062276055 |
| CH20220713 | 0.0912758557192 |
| CH20220714 | 0.417679232639 |
| CH20220715 | 0.660843216725 |
| CH20220716 | 0.141588350367 |
| CH20220717 | 0.412075832388 |
| CH20220718 | 0.237752601103 |
| CH20220719 | 0.10555770861 |
| CH20220720 | 0.638126717424 |
| CH20220721 | 0.241709137535 |
| CH20220722 | 0.404141116886 |
| CH20220723 | 0.41223291893 |
| CH20220724 | 0.399267935669 |
| CH20220725 | 0.180258857865 |
| CH20220726 | 0.374914708376 |
| CH20220727 | 0.294616498881 |
| CH20220728 | 0.563026402122 |
| CH20220729 | 0.939740683873 |
| CH20220730 | 0.770668949141 |
| CH20220731 | 0.948886115379 |
| CH20220732 | 0.511177375669 |
| CH20220733 | 0.20110786375 |
| CH20220734 | 0.330475788096 |
| CH20220735 | 0.326564291165 |
| HM20220701 | 0.847102367185 |
| HM20220702 | 0.168249925741 |
| HM20220703 | 0.849190055264 |
| HM20220704 | 0.949188355923 |
| LC20220701 | 0.0 |
| LC20220702 | 0.0045170970184 |
| LC20220703 | 0.966121557556 |
| LC20220704 | 0.662692223995 |
| LC20220705 | 0.429420085142 |
| LC20220706 | 0.20419726068 |
| LC20220707 | 0.740403949358 |
| LC20220708 | 0.639764656064 |
| LC20220709 | 0.859493073048 |
| LC20220710 | 0.0641943825407 |
| LC20220711 | 0.123485414882 |
| LC20220712 | 0.339345904297 |
| LC20220713 | 0.784183249457 |
| LC20220714 | 0.187880151041 |
| LC20220715 | 0.134895552301 |
| LC20220716 | 0.282443238009 |
| LC20220717 | 0.507046500875 |
| LC20220718 | 0.6214511652 |
| LC20220719 | 0.333070780482 |
| LC20220720 | 0.564694437519 |
| LC20220721 | 0.145994454657 |
| LC20220722 | 0.0492278778174 |
| LC20220723 | 0.0115858386728 |
| LC20220724 | 0.172425534188 |
| LC20220725 | 0.717408681438 |
| LC20220726 | 0.315299283114 |
| LC20220727 | 0.235199011986 |
| LC20220728 | 0.16559216847 |
| LC20220729 | 0.890526617058 |
| LC20220730 | 0.0933596885385 |
| LC20220731 | 0.107366417672 |
| LL20220701 | 0.408524963559 |
| LL20220702 | 0.0886002687153 |
| LL20220703 | 0.0182524268851 |
| LL20220704 | 0.901384941024 |
| LL20220705 | 0.931361429754 |
| LL20220706 | 0.278809332331 |
| LL20220707 | 0.501864988998 |
| LL20220708 | 0.00806748359774 |
| LL20220709 | 0.339807405964 |
| LL20220710 | 0.0513462843931 |
| LL20220711 | 0.729310005897 |
| LL20220712 | 0.472566378909 |
| LL20220713 | 0.0823928667519 |
| LL20220714 | 0.868520886068 |
| LL20220715 | 0.0395102245208 |
| LL20220716 | 0.216726229769 |
| LL20220717 | 0.69880557739 |
| LL20220718 | 0.938873597043 |
| LL20220719 | 0.32388126634 |
| LL20220720 | 0.78250778888 |
| LL20220721 | 0.551786112111 |
| LL20220722 | 0.53546658833 |
| LL20220723 | 0.601923524798 |
| LL20220724 | 0.711152561346 |
| LL20220725 | 0.778274155512 |
| LL20220726 | 0.813239917539 |
| LL20220727 | 0.957026664431 |
| LL20220728 | 0.388454410431 |
| LL20220729 | 0.000845955526909 |
| LL20220730 | 0.884800455074 |
| LL20220731 | 0.215726353819 |
| LL20220732 | 0.304792516999 |
| LL20220733 | 0.644258511252 |
| LL20220734 | 0.333757151939 |
| LL20220735 | 0.537318827633 |
| LL20220736 | 0.50924632661 |
| LL20220737 | 0.82884025849 |
| LL20220738 | 0.26295377429 |
| LL20220739 | 0.209893350265 |
| LL20220740 | 0.828672144785 |
| LL20220741 | 0.29309963844 |
| PF20220701 | 0.548545649727 |
| PF20220702 | 0.0503648098878 |
| PF20220703 | 0.00567037181926 |
| PF20220704 | 0.427652147347 |
| PF20220705 | 0.0569633416824 |
| PF20220706 | 0.193452514131 |
| PF20220707 | 0.0581581263143 |
| PF20220708 | 0.0468250605474 |
| PF20220709 | 0.152867710275 |
| PF20220710 | 0.0131858917772 |
| PF20220711 | 0.954378323522 |
| PF20220712 | 0.951127380688 |
| PF20220713 | 0.851438483231 |
| PF20220714 | 0.798039460681 |
| PF20220715 | 0.370610857641 |
| PF20220716 | 0.0113911859132 |
| PN20220701 | 0.447589090802 |
| PN20220702 | 0.0380474778762 |
| PN20220703 | 0.250223932356 |
| PN20220704 | 0.319403919659 |
| PN20220705 | 0.0792171327674 |
| PN20220706 | 0.44087464607 |
| PN20220707 | 0.413588229697 |
| PN20220708 | 0.527657990967 |
| PN20220709 | 0.0449023048169 |
| PN20220710 | 0.0109517464405 |
| PN20220711 | 0.00742703291188 |
| PT20220701 | 0.27372430704 |
| PT20220702 | 0.0254423853409 |
| PT20220703 | 0.353008777136 |
| PT20220704 | 0.0222836510345 |
| PT20220705 | 0.0996345440541 |
| PT20220706 | 0.607895875842 |
| PV20220701 | 0.153664305228 |
| PV20220702 | 0.317926599102 |
| PV20220703 | 0.0456707904425 |
| PV20220704 | 0.0788893755754 |
| SC20220701 | 0.199836631698 |
| SC20220702 | 0.0219603870204 |
| SC20220703 | 0.102379411789 |
| SC20220704 | 0.0 |
| SC20220705 | 0.343717428634 |
| SC20220706 | 0.199824424594 |
| SC20220707 | 0.102428842563 |
| SC20220708 | 0.806043820273 |
| SC20220709 | 0.318765751671 |
| SK20220701 | 0.104063947904 |
| SK20220702 | 0.0639680873082 |
| SK20220703 | 0.608472988394 |
| SK20220704 | 0.289577996663 |
| SK20220705 | 0.293624775765 |
| SM20220701 | 0.857619583764 |
| SM20220702 | 0.198226295741 |
| XA20220701 | 0.170354289684 |
| XA20220702 | 0.139241300205 |
| XA20220703 | 0.0997031147272 |
| XA20220704 | 0.108237938748 |
| XA20220705 | 0.0154037449997 |
| XA20220706 | 0.175966481828 |
| XA20220707 | 0.0617731859037 |
| XA20220708 | 0.0947089505241 |
| XA20220709 | 0.122726044707 |
| XA20220710 | 0.18858467471 |
| XA20220711 | 0.0856252188142 |
| XA20220712 | 0.205731487733 |
| XA20220713 | 0.248258857842 |
| XA20220714 | 0.129927517803 |
| WHW20220701 | 0.159635425862 |
| WHW20220702A | 0.318505089782 |
| WHW20220702B | 0.248912624538 |
| WHW20220702C | 0.191812495177 |
| WHW20220702D | 0.234163850471 |
| WHW20220702E | 0.190315358837 |
| WHW20220702F | 0.209419233939 |
| WHW20220702G | 0.242573730685 |
| WHW20220702H | 0.429884296587 |
| WHW20220703 | 0.211166512926 |
| WHW20220704 | 0.211154918266 |
| WHW20220705 | 0.182457291129 |
| WHW20220706 | 0.20874765914 |
| WHW20220707 | 0.185501246311 |
| WHW20220708 | 0.196584297598 |
| WHW20220709 | 0.187248425189 |
| WHW20220710 | 0.174754446546 |
| WHW20220711 | 0.182642387004 |
| WHW20220712 | 0.171313962005 |
| WHW20220713 | 0.166791560021 |
| WHW20220714A | 0.218741930563 |
| WHW20220714B | 0.171002987115 |
| WHW20220714C | 0.142722530249 |
| WHW20220714D | 0.133759473362 |
| WHW20220714E | 0.14699668709 |
| WHW20220714F | 0.14876888927 |
| WHW20220714G | 0.149451082905 |
| WHW20220714H | 0.174143034211 |
| WHW20220715 | 0.162975354673 |
| WHW20220716 | 0.125956687373 |
### Chart: Percent of the microbes with the phenotype of Contains Mobile Elements in each sample
| Category | Contains_Mobile_Elements |
|---|---|
| AN20220701 | 0.41366286343 |
| AN20220702 | 0.243196386473 |
| AN20220703 | 0.517207696551 |
| AN20220704 | 0.403291446887 |
| AN20220705 | 0.719461997797 |
| CB20220701 | 0.0100073955096 |
| CB20220702 | 0.0726449917375 |
| CB20220703 | 0.592054289779 |
| CB20220704 | 0.237689728997 |
| CB20220705 | 0.891830770868 |
| CB20220706 | 0.603575439751 |
| CB20220707 | 0.217772624303 |
| CB20220708 | 0.156878287814 |
| CB20220709 | 0.479823788546 |
| CB20220710 | 0.849286977462 |
| CB20220711 | 0.975874695559 |
| CB20220712 | 0.626168566668 |
| CB20220713 | 0.583573306029 |
| CB20220714 | 0.400957002812 |
| CB20220715 | 0.961948528318 |
| CB20220716 | 0.915081916204 |
| CB20220717 | 0.53587968812 |
| CB20220718 | 0.10973311499 |
| CB20220719 | 0.959389484708 |
| CB20220720 | 0.291436567408 |
| CB20220721 | 0.0718416662208 |
| CB20220722 | 0.284546373364 |
| CB20220723 | 0.0881749081588 |
| CB20220724 | 0.996816312608 |
| CB20220725 | 0.00355871886121 |
| CB20220726 | 0.42061952036 |
| CB20220727 | 0.53561606862 |
| CB20220728 | 0.123039165082 |
| CB20220729 | 0.0167611523015 |
| CB20220730 | 0.0804547303726 |
| CB20220731 | 0.043585855587 |
| CH20220701 | 0.170954096196 |
| CH20220702 | 0.153674861838 |
| CH20220703 | 0.304849518659 |
| CH20220704 | 0.203015867774 |
| CH20220705 | 0.19282760277 |
| CH20220706 | 0.012393395374 |
| CH20220707 | 0.481093117039 |
| CH20220708 | 0.394275094407 |
| CH20220709 | 0.0486415196811 |
| CH20220710 | 0.1174664816 |
| CH20220711 | 0.157338273353 |
| CH20220712 | 0.177844813837 |
| CH20220713 | 0.434168760278 |
| CH20220714 | 0.216920400468 |
| CH20220715 | 0.0813518882582 |
| CH20220716 | 0.495897986855 |
| CH20220717 | 0.218288024081 |
| CH20220718 | 0.35819149693 |
| CH20220719 | 0.105429634201 |
| CH20220720 | 0.456446580702 |
| CH20220721 | 0.467720182674 |
| CH20220722 | 0.211072899201 |
| CH20220723 | 0.145160457235 |
| CH20220724 | 0.264726502912 |
| CH20220725 | 0.105979945748 |
| CH20220726 | 0.396349677743 |
| CH20220727 | 0.0873083843101 |
| CH20220728 | 0.354400925139 |
| CH20220729 | 0.0309156491433 |
| CH20220730 | 0.126929406091 |
| CH20220731 | 0.0119579253201 |
| CH20220732 | 0.360231940082 |
| CH20220733 | 0.456918941085 |
| CH20220734 | 0.244857924936 |
| CH20220735 | 0.238577675833 |
| HM20220701 | 0.364913158842 |
| HM20220702 | 0.344643382908 |
| HM20220703 | 0.401558241673 |
| HM20220704 | 0.932992297149 |
| LC20220701 | 0.0 |
| LC20220702 | 0.00284114229426 |
| LC20220703 | 0.953347549586 |
| LC20220704 | 0.312831858407 |
| LC20220705 | 0.39953809494 |
| LC20220706 | 0.159567968603 |
| LC20220707 | 0.0666380146793 |
| LC20220708 | 0.639764656064 |
| LC20220709 | 0.198713361575 |
| LC20220710 | 0.0467875590739 |
| LC20220711 | 0.123485414882 |
| LC20220712 | 0.332179660505 |
| LC20220713 | 0.7535755659 |
| LC20220714 | 0.244320250387 |
| LC20220715 | 0.176982964619 |
| LC20220716 | 0.25071088099 |
| LC20220717 | 0.475168781697 |
| LC20220718 | 0.615318213564 |
| LC20220719 | 0.209988589914 |
| LC20220720 | 0.564415267666 |
| LC20220721 | 0.129250363088 |
| LC20220722 | 0.0409084535392 |
| LC20220723 | 0.00683132986832 |
| LC20220724 | 0.0319277490511 |
| LC20220725 | 0.647434458106 |
| LC20220726 | 0.314414411884 |
| LC20220727 | 0.208095255177 |
| LC20220728 | 0.0659957566112 |
| LC20220729 | 0.703817655664 |
| LC20220730 | 0.112197784062 |
| LC20220731 | 0.0514252081981 |
| LL20220701 | 0.463706523168 |
| LL20220702 | 0.0251283287973 |
| LL20220703 | 0.0243077855382 |
| LL20220704 | 0.872908894584 |
| LL20220705 | 0.148322259853 |
| LL20220706 | 0.356069823347 |
| LL20220707 | 0.216542566221 |
| LL20220708 | 0.0024623644716 |
| LL20220709 | 0.0750706732889 |
| LL20220710 | 0.0538751002225 |
| LL20220711 | 0.31030076666 |
| LL20220712 | 0.487202582702 |
| LL20220713 | 0.0592870018335 |
| LL20220714 | 0.841210312616 |
| LL20220715 | 0.0352699190034 |
| LL20220716 | 0.209897339009 |
| LL20220717 | 0.320460681453 |
| LL20220718 | 0.0325205145825 |
| LL20220719 | 0.305811487037 |
| LL20220720 | 0.488386747071 |
| LL20220721 | 0.914453841276 |
| LL20220722 | 0.136747239948 |
| LL20220723 | 0.130791995664 |
| LL20220724 | 0.567326689767 |
| LL20220725 | 0.876855880692 |
| LL20220726 | 0.512253555343 |
| LL20220727 | 0.937432796362 |
| LL20220728 | 0.409379477136 |
| LL20220729 | 0.000422977763455 |
| LL20220730 | 0.842642938496 |
| LL20220731 | 0.0708349295902 |
| LL20220732 | 0.308466635859 |
| LL20220733 | 0.595787651471 |
| LL20220734 | 0.267005721551 |
| LL20220735 | 0.213249461946 |
| LL20220736 | 0.501201184424 |
| LL20220737 | 0.844364048999 |
| LL20220738 | 0.169553287302 |
| LL20220739 | 0.182854252537 |
| LL20220740 | 0.263166520652 |
| LL20220741 | 0.241693024209 |
| PF20220701 | 0.200299014046 |
| PF20220702 | 0.670739831722 |
| PF20220703 | 0.96975883027 |
| PF20220704 | 0.200181156755 |
| PF20220705 | 0.0329360726389 |
| PF20220706 | 0.0543666936712 |
| PF20220707 | 0.0857767293129 |
| PF20220708 | 0.0185321457687 |
| PF20220709 | 0.168671584795 |
| PF20220710 | 0.0129406605997 |
| PF20220711 | 0.0323552782904 |
| PF20220712 | 0.043851715711 |
| PF20220713 | 0.288613707518 |
| PF20220714 | 0.793647367963 |
| PF20220715 | 0.270231068072 |
| PF20220716 | 0.00520344102225 |
| PN20220701 | 0.412907841171 |
| PN20220702 | 0.0435245981496 |
| PN20220703 | 0.351886229728 |
| PN20220704 | 0.314838086873 |
| PN20220705 | 0.0621993226619 |
| PN20220706 | 0.665229598721 |
| PN20220707 | 0.692360164859 |
| PN20220708 | 0.16783945791 |
| PN20220709 | 0.00892706111795 |
| PN20220710 | 0.0070468462264 |
| PN20220711 | 0.0123686084215 |
| PT20220701 | 0.210121672074 |
| PT20220702 | 0.0170751662324 |
| PT20220703 | 0.346090008958 |
| PT20220704 | 0.285935120438 |
| PT20220705 | 0.0286738155458 |
| PT20220706 | 0.59831612358 |
| PV20220701 | 0.105762369767 |
| PV20220702 | 0.0783771748333 |
| PV20220703 | 0.0455122296609 |
| PV20220704 | 0.183629672955 |
| SC20220701 | 0.20620056709 |
| SC20220702 | 0.0113526559766 |
| SC20220703 | 0.0975883229953 |
| SC20220704 | 0.0 |
| SC20220705 | 0.343986957104 |
| SC20220706 | 0.199196835907 |
| SC20220707 | 0.0651641895439 |
| SC20220708 | 0.077660819905 |
| SC20220709 | 0.127015512689 |
| SK20220701 | 0.104806428711 |
| SK20220702 | 0.0714104128293 |
| SK20220703 | 0.0951574638605 |
| SK20220704 | 0.0797001296645 |
| SK20220705 | 0.100959473458 |
| SM20220701 | 0.0914967815656 |
| SM20220702 | 0.461428395889 |
| XA20220701 | 0.403021882598 |
| XA20220702 | 0.432481098244 |
| XA20220703 | 0.502039160092 |
| XA20220704 | 0.305251134898 |
| XA20220705 | 0.899406919006 |
| XA20220706 | 0.393438770687 |
| XA20220707 | 0.237253294998 |
| XA20220708 | 0.163456577278 |
| XA20220709 | 0.220174187669 |
| XA20220710 | 0.422625834422 |
| XA20220711 | 0.5279262394 |
| XA20220712 | 0.61517699335 |
| XA20220713 | 0.427301537897 |
| XA20220714 | 0.491133814317 |
| WHW20220701 | 0.725737609081 |
| WHW20220702A | 0.610132081212 |
| WHW20220702B | 0.664283808117 |
| WHW20220702C | 0.699418104997 |
| WHW20220702D | 0.650305622707 |
| WHW20220702E | 0.699498142188 |
| WHW20220702F | 0.683170935016 |
| WHW20220702G | 0.669325073048 |
| WHW20220702H | 0.505125421047 |
| WHW20220703 | 0.631867190469 |
| WHW20220704 | 0.621464925321 |
| WHW20220705 | 0.660325301415 |
| WHW20220706 | 0.606676041896 |
| WHW20220707 | 0.619552760508 |
| WHW20220708 | 0.660037149197 |
| WHW20220709 | 0.665670565775 |
| WHW20220710 | 0.703352419309 |
| WHW20220711 | 0.688606465232 |
| WHW20220712 | 0.721100265469 |
| WHW20220713 | 0.67863802556 |
| WHW20220714A | 0.608957513281 |
| WHW20220714B | 0.676237701845 |
| WHW20220714C | 0.682522601262 |
| WHW20220714D | 0.682021320981 |
| WHW20220714E | 0.658206649338 |
| WHW20220714F | 0.525738117394 |
| WHW20220714G | 0.534575323677 |
| WHW20220714H | 0.551529026097 |
| WHW20220715 | 0.579949284142 |
| WHW20220716 | 0.604918145314 |
### Chart: Percent of the microbes with the phenotype of Stress Tolerant in each sample
| Category | Stress_Tolerant |
|---|---|
| AN20220701 | 0.00857299210448 |
| AN20220702 | 0.316482331777 |
| AN20220703 | 0.0211605728715 |
| AN20220704 | 0.0186970005281 |
| AN20220705 | 0.00608730940924 |
| CB20220701 | 0.120620911465 |
| CB20220702 | 0.670674793978 |
| CB20220703 | 0.615091065973 |
| CB20220704 | 0.158821454526 |
| CB20220705 | 0.874765158888 |
| CB20220706 | 0.700486902486 |
| CB20220707 | 0.234575697707 |
| CB20220708 | 0.2707881583 |
| CB20220709 | 0.801762114537 |
| CB20220710 | 0.880761560748 |
| CB20220711 | 0.906990634611 |
| CB20220712 | 0.505185137327 |
| CB20220713 | 0.301325523129 |
| CB20220714 | 0.448420245129 |
| CB20220715 | 0.941810354364 |
| CB20220716 | 0.956764474486 |
| CB20220717 | 0.249494003849 |
| CB20220718 | 0.857358275698 |
| CB20220719 | 0.996858212284 |
| CB20220720 | 0.6033320107 |
| CB20220721 | 0.535315261855 |
| CB20220722 | 0.559802253681 |
| CB20220723 | 0.764833974079 |
| CB20220724 | 1.0 |
| CB20220725 | 0.986088644452 |
| CB20220726 | 0.190185006112 |
| CB20220727 | 0.0728590191528 |
| CB20220728 | 0.866834517455 |
| CB20220729 | 0.963235388101 |
| CB20220730 | 0.668983376002 |
| CB20220731 | 0.148407547119 |
| CH20220701 | 0.287922429301 |
| CH20220702 | 0.600586681361 |
| CH20220703 | 0.318648093363 |
| CH20220704 | 0.282160725195 |
| CH20220705 | 0.489045612417 |
| CH20220706 | 0.0544234804036 |
| CH20220707 | 0.0278969168009 |
| CH20220708 | 0.227255316765 |
| CH20220709 | 0.506380883025 |
| CH20220710 | 0.719598328657 |
| CH20220711 | 0.55063252109 |
| CH20220712 | 0.347858742273 |
| CH20220713 | 0.0130883411519 |
| CH20220714 | 0.362574207829 |
| CH20220715 | 0.603394407241 |
| CH20220716 | 0.0387687940039 |
| CH20220717 | 0.372380454038 |
| CH20220718 | 0.140539710429 |
| CH20220719 | 0.0961578231564 |
| CH20220720 | 0.0853019440199 |
| CH20220721 | 0.103997409435 |
| CH20220722 | 0.33846962214 |
| CH20220723 | 0.360529811499 |
| CH20220724 | 0.351381009629 |
| CH20220725 | 0.152096203576 |
| CH20220726 | 0.303227944601 |
| CH20220727 | 0.281275412711 |
| CH20220728 | 0.501472719321 |
| CH20220729 | 0.932282908045 |
| CH20220730 | 0.731640373692 |
| CH20220731 | 0.941734198241 |
| CH20220732 | 0.455674097057 |
| CH20220733 | 0.0734236374643 |
| CH20220734 | 0.256438084567 |
| CH20220735 | 0.245137211205 |
| HM20220701 | 0.829520013256 |
| HM20220702 | 0.0599729230298 |
| HM20220703 | 0.695817562832 |
| HM20220704 | 0.948026793805 |
| LC20220701 | 0.0 |
| LC20220702 | 0.00111730314943 |
| LC20220703 | 0.0139527770666 |
| LC20220704 | 0.386850790657 |
| LC20220705 | 0.0413125590179 |
| LC20220706 | 0.00125858127684 |
| LC20220707 | 0.683072944363 |
| LC20220708 | 0.00259747063112 |
| LC20220709 | 0.724181360202 |
| LC20220710 | 0.0526926941903 |
| LC20220711 | 0.114681676055 |
| LC20220712 | 0.321183955185 |
| LC20220713 | 0.139721467876 |
| LC20220714 | 0.0102739660749 |
| LC20220715 | 0.0971247976567 |
| LC20220716 | 0.248800352643 |
| LC20220717 | 0.465218721868 |
| LC20220718 | 0.616426770491 |
| LC20220719 | 0.294076059931 |
| LC20220720 | 0.0110427186003 |
| LC20220721 | 0.0437790935836 |
| LC20220722 | 0.0270717707245 |
| LC20220723 | 0.00731524933951 |
| LC20220724 | 0.143146608271 |
| LC20220725 | 0.692512192274 |
| LC20220726 | 0.0535838689379 |
| LC20220727 | 0.178508652806 |
| LC20220728 | 0.141758803295 |
| LC20220729 | 0.224087854311 |
| LC20220730 | 0.0112772272524 |
| LC20220731 | 0.0577784134039 |
| LL20220701 | 0.0111237125201 |
| LL20220702 | 0.0178776082039 |
| LL20220703 | 0.00647631941516 |
| LL20220704 | 0.629921032312 |
| LL20220705 | 0.353426081087 |
| LL20220706 | 0.0160711109559 |
| LL20220707 | 0.207148824389 |
| LL20220708 | 0.00414714016269 |
| LL20220709 | 0.254088421795 |
| LL20220710 | 0.0374415716833 |
| LL20220711 | 0.462355022607 |
| LL20220712 | 0.0449808479186 |
| LL20220713 | 0.0206990039894 |
| LL20220714 | 0.0937977822607 |
| LL20220715 | 0.00367493144839 |
| LL20220716 | 0.0176640641005 |
| LL20220717 | 0.383103790028 |
| LL20220718 | 0.914777616879 |
| LL20220719 | 0.258263532024 |
| LL20220720 | 0.303321626427 |
| LL20220721 | 0.336068839648 |
| LL20220722 | 0.356372445299 |
| LL20220723 | 0.50162409862 |
| LL20220724 | 0.581957937642 |
| LL20220725 | 0.0657654366094 |
| LL20220726 | 0.429092302977 |
| LL20220727 | 0.925659212201 |
| LL20220728 | 0.121186630039 |
| LL20220729 | 0.000422977763455 |
| LL20220730 | 0.0571651149334 |
| LL20220731 | 0.0580759519386 |
| LL20220732 | 0.143431772705 |
| LL20220733 | 0.0484708597807 |
| LL20220734 | 0.0534011443102 |
| LL20220735 | 0.410427392245 |
| LL20220736 | 0.00960947538969 |
| LL20220737 | 0.711920906382 |
| LL20220738 | 0.127352399451 |
| LL20220739 | 0.0276579029498 |
| LL20220740 | 0.709682894315 |
| LL20220741 | 0.00726571259863 |
| PF20220701 | 0.462447789364 |
| PF20220702 | 0.00577405751364 |
| PF20220703 | 0.0023569617803 |
| PF20220704 | 0.0573582343723 |
| PF20220705 | 0.0454055622106 |
| PF20220706 | 0.159436096327 |
| PF20220707 | 0.0257388027945 |
| PF20220708 | 0.0406955903704 |
| PF20220709 | 0.141543843797 |
| PF20220710 | 0.00537173055574 |
| PF20220711 | 0.290769930184 |
| PF20220712 | 0.0613796376709 |
| PF20220713 | 0.564603858619 |
| PF20220714 | 0.328933668041 |
| PF20220715 | 0.307010800689 |
| PF20220716 | 0.00144545410635 |
| PN20220701 | 0.0671131040179 |
| PN20220702 | 0.000351338757514 |
| PN20220703 | 0.13150930519 |
| PN20220704 | 0.317212319921 |
| PN20220705 | 0.0128330349131 |
| PN20220706 | 0.0317569551484 |
| PN20220707 | 0.347120634734 |
| PN20220708 | 0.506358328699 |
| PN20220709 | 0.035081376721 |
| PN20220710 | 0.00672929798033 |
| PN20220711 | 0.00168700945092 |
| PT20220701 | 0.218648618739 |
| PT20220702 | 0.0130751825547 |
| PT20220703 | 0.262687403464 |
| PT20220704 | 0.000658305791269 |
| PT20220705 | 0.0767785249564 |
| PT20220706 | 0.50650535529 |
| PV20220701 | 0.0866843237372 |
| PV20220702 | 0.175658549266 |
| PV20220703 | 0.03813386796 |
| PV20220704 | 0.0190283347715 |
| SC20220701 | 0.1846762446 |
| SC20220702 | 0.0205301311494 |
| SC20220703 | 0.0977921991142 |
| SC20220704 | 0.0 |
| SC20220705 | 0.343467865236 |
| SC20220706 | 0.198360050992 |
| SC20220707 | 0.0816551451949 |
| SC20220708 | 0.765702025182 |
| SC20220709 | 0.273030493171 |
| SK20220701 | 0.0730295777206 |
| SK20220702 | 0.0418660343604 |
| SK20220703 | 0.593631318016 |
| SK20220704 | 0.174936312042 |
| SK20220705 | 0.177181501975 |
| SM20220701 | 0.381763752772 |
| SM20220702 | 0.0648341041025 |
| XA20220701 | 0.0905696422369 |
| XA20220702 | 0.0154977021691 |
| XA20220703 | 0.0186965811966 |
| XA20220704 | 0.00881686425672 |
| XA20220705 | 0.00145066247829 |
| XA20220706 | 0.0292604020167 |
| XA20220707 | 0.0477865405543 |
| XA20220708 | 0.0536030937907 |
| XA20220709 | 0.0496444259775 |
| XA20220710 | 0.100728069836 |
| XA20220711 | 0.010791306031 |
| XA20220712 | 0.00620371978208 |
| XA20220713 | 0.0784450881297 |
| XA20220714 | 0.0277965545859 |
| WHW20220701 | 0.0785675090479 |
| WHW20220702A | 0.2113921973 |
| WHW20220702B | 0.139720425565 |
| WHW20220702C | 0.101159798958 |
| WHW20220702D | 0.124669319984 |
| WHW20220702E | 0.102125856443 |
| WHW20220702F | 0.121977528409 |
| WHW20220702G | 0.156338703194 |
| WHW20220702H | 0.367039966417 |
| WHW20220703 | 0.0987039873066 |
| WHW20220704 | 0.10467327762 |
| WHW20220705 | 0.084448920596 |
| WHW20220706 | 0.0869376403076 |
| WHW20220707 | 0.0769145918689 |
| WHW20220708 | 0.0952710662687 |
| WHW20220709 | 0.0854080490589 |
| WHW20220710 | 0.0852009537019 |
| WHW20220711 | 0.0859742614984 |
| WHW20220712 | 0.0910621156179 |
| WHW20220713 | 0.0854845386576 |
| WHW20220714A | 0.0976842928408 |
| WHW20220714B | 0.0784563852174 |
| WHW20220714C | 0.0615355201704 |
| WHW20220714D | 0.05683968268 |
| WHW20220714E | 0.0620207533322 |
| WHW20220714F | 0.072567756722 |
| WHW20220714G | 0.073363602915 |
| WHW20220714H | 0.0864693889991 |
| WHW20220715 | 0.0752960707077 |
| WHW20220716 | 0.055891134983 |
