# Supplementary material for: Effectiveness assessment of using water environmental microHI to predict the health status of wild fish
Source: Front Microbiol. 2024 Jan 11;14:1293342. doi: 10.3389/fmicb.2023.1293342 (PMC10808811; doi:10.3389/fmicb.2023.1293342)
Supplement: Supplementary file 2 [file Data_Sheet_1.ZIP › Supplementary Figure S6 Hcluster species level.pdf]

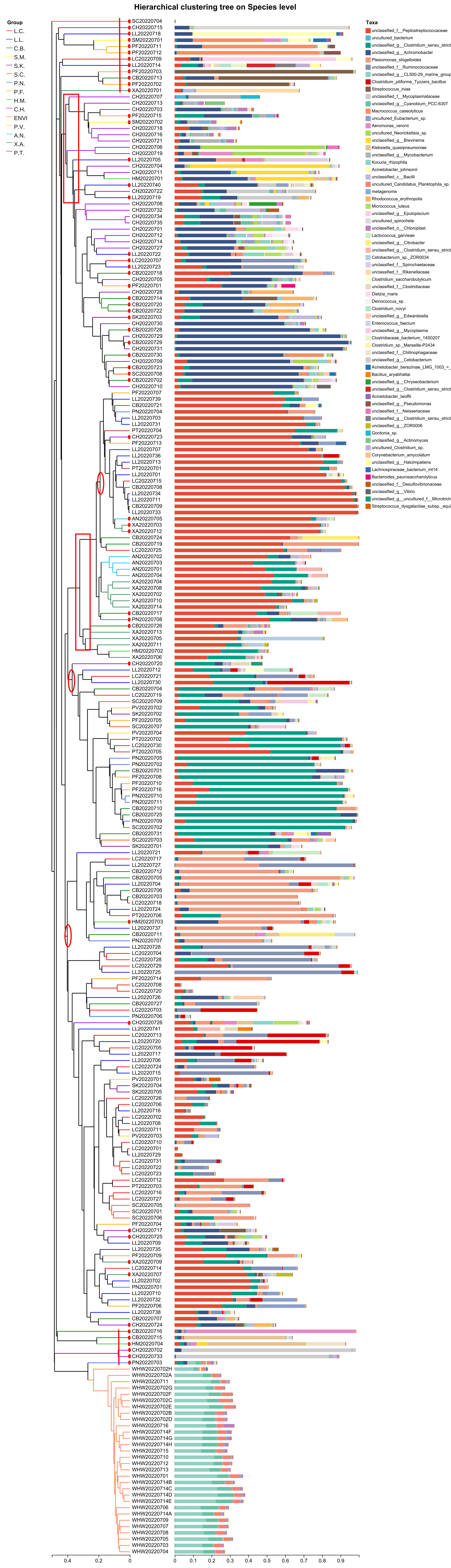
Hierarchical clustering tree on Species level
Group
L.C.
L.L.
C.B.
S.M.
S.K.
S.C.
P.N.
P.F.
H.M.
C.H.
ENVI
P.V.
A.N.
X.A.
P.T.
Taxa
unclassified_f__Peptostreptococcaceae
uncultured_bacterium
unclassified_g__Clostridium_sensu_stricto_1
unclassified_g__Achromobacter
Plesiomonas_shigelloides
unclassified_f__Ruminococcaceae
unclassified_g__CL500-29_marine_group
Clostridium_piliforme_Tyzzers_bacillus
Streptococcus_iniae
unclassified_f__Mycoplasmataceae
unclassified_g__Cyanobium_PCC-6307
Macrococcus_caseolyticus
uncultured_Eubacterium_sp.
Aeromonas_veronii
uncultured_Neorickettsia_sp.
unclassified_g__Brevinema
Klebsiella_quasipneumoniae
unclassified_g__Mycobacterium
Kocuria_rhizophila
Acinetobacter_johnsonii
unclassified_c__Bacilli
uncultured_Candidatus_Planktophila_sp.
metagenome
Rhodococcus_erythropolis
Micrococcus_luteus
unclassified_g__Epulopiscium
unclassified_o__Chloroplast
Lactococcus_garvieae
unclassified_g__Citrobacter
unclassified_g__Clostridium_sensu_stricto_13
Cetobacterium_sp._ZOR0034
unclassified_f__Spirochaetaceae
unclassified_f__Rikenellaceae
Clostridium_saccharobutylicum
unclassified_f__Clostridiaceae
Dietzia_maris
Deinococcus_sp.
Clostridium_novyi
unclassified_g__Edwardsiella
Enterococcus_faecium
unclassified_g__Mycoplasma
Clostridiaceae_bacterium_14S0207
Clostridium_sp._Marseille-P2434
unclassified_f__Chitinophagaceae
unclassified_g__Cetobacterium
Acinetobacter_bereziniae_LMG_1003_=_CIP_70.12
Bacillus_aryabhattai
unclassified_g__Chryseobacterium
unclassified_g__Clostridium_sensu_stricto_7
Acinetobacter_lwoffii
unclassified_g__Pseudomonas
unclassified_f__Neisseriaceae
unclassified_g__Clostridium_sensu_stricto_2
unclassified_g__ZOR0006
Gordonia_sp.
unclassified_g__Actinomyces
uncultured_Clostridium_sp.
Corynebacterium_amycolatum
unclassified_g__Haloimpatiens
Lachnospiraceae_bacterium_mt14
Bacteroides_paurosaccharolyticus
unclassified_f__Desulfovibrionaceae
unclassified_g__Vibrio
unclassified_g__uncultured_f__Microtrichaceae
Streptococcus_dysgalactiae_subsp._equisimilis
0.4
0.2
0
0
0.1
0.2
0.3
0.4
0.5
0.6
0.7
0.8
0.9
