# Supplementary material for: Effectiveness assessment of using water environmental microHI to predict the health status of wild fish
Source: Front Microbiol. 2024 Jan 11;14:1293342. doi: 10.3389/fmicb.2023.1293342 (PMC10808811; doi:10.3389/fmicb.2023.1293342)
Supplement: Supplementary file 2 [file Data_Sheet_1.ZIP › Supplementary Table S1 sampling details.docx]

Supplementary Table S1 Details for eDNA sampling and corresponding hydrological conditions

| Date | eDNA samples | Temperature /℃ | Water level/m | Discharge /m³/s |
| --- | --- | --- | --- | --- |
| 2022.6.27 | WHW01 | 26.6 | 24.63 | 40100 |
| 2022.6.28 | WHW02(A/B/C/D/E/F/G/H) | 26.8 | 24.65 | 40200 |
| 2022.6.29 | WHW03 | 26.6 | 24.61 | 37900 |
| 2022.6.30 | WHW04 | 27.1 | 24.50 | 37400 |
| 2022.7.1 | WHW05 | 27.1 | 24.43 | 37100 |
| 2022.7.2 | WHW06 | 27.1 | 24.34 | 36800 |
| 2022.7.4 | WHW07 | 27.1 | 24.04 | 36700 |
| 2022.7.5 | WHW08 | 27.4 | 24.00 | 36500 |
| 2022.7.6 | WHW09 | 27.3 | 24.15 | 37200 |
| 2022.7.7 | WHW10 | 27.6 | 24.28 | 37800 |
| 2022.7.8 | WHW11 | 27.6 | 24.37 | 38200 |
| 2022.7.9 | WHW12 | 27.9 | 24.27 | 37700 |
| 2022.7.10 | WHW13 | 28.3 | 24.12 | 37000 |
| 2022.7.12 | WHW14(A/B/C/D/E/F/G/H) | 29.3 | 23.54 | 34800 |
| 2022.7.13 | WHW15 | 29.4 | 23.29 | 34000 |
| 2022.7.14 | WHW16 | 29.6 | 22.96 | 32800 |

Note: We respectively processed the transection sampling in 28 June and 12 July, 2022. Eight sampling sites (A/B/C/D/E/F/G/H) were approximately equally set in the transection of Yangtze River.
