# Supplementary material for: Effectiveness assessment of using water environmental microHI to predict the health status of wild fish
Source: Front Microbiol. 2024 Jan 11;14:1293342. doi: 10.3389/fmicb.2023.1293342 (PMC10808811; doi:10.3389/fmicb.2023.1293342)
Supplement: Supplementary file 2 [file Data_Sheet_1.ZIP › Supplementary Table S2 microHI base values.docx]

Supplementary Table S2 The base value of the fish species gut microHI

| Group label | Species | Gut microHI base value | Habitats | Diets |
| --- | --- | --- | --- | --- |
| *X.A.* | *Xenocypris argentea* | 0.85 | Bottom | Bottom attached algae, phytoclasts |
| *P.N.* | *Pelteobagrus nitidus* | 0.83 | Bottom | Little fishes, shrimps, aquatic insects |
| *S.C.* | *Siniperca chuatsi* | 0.82 | Bottom | Fishes, shrimps |
| *P.F.* | *Pelteobagrus fulvidraco* | 0.81 | Bottom | Little fishes, shrimps, molluscs, aquatic insects |
| *P.V.* | *Pelteobagrus vachelli* | 0.74 | Bottom | Aquatic insects, Oligochaeta, shrimps, small mollusks, little fishes |
| *C.H.* | *Coreius heterodon* | 0.70 | Bottom | Small mollusks, fish eggs and larvae, phytoclasts |
| *L.C.* | *Leiocassis crassilabris* | 0.69 | Bottom | Oligochaeta, small mollusks, shrimps, little fishes |
| *L.L.* | *Leiocassis longirostris* | 0.67 | Bottom | Little fishes, shrimps, aquatic insects |
| *S.A.* | *Silurus asotus* | 0.67 | Bottom | Shrimps, little fishes |
| *H.M.* | *Hypophthalmichthys molitrix* | 0.64 | Pelagic | Phytoplankton, zooplankton |
| *C.B.* | *Coilia brachygnathus* | 0.63 | Pelagic | Little fishes, shrimps |

Note: The base value of the species gut microHI is indicated by the average gut microHI of healthy individuals in each species
