# Supplementary figures and images for: Effectiveness assessment of using water environmental microHI to predict the health status of wild fish
Source: Front Microbiol. 2024 Jan 11;14:1293342. doi: 10.3389/fmicb.2023.1293342 (PMC10808811; doi:10.3389/fmicb.2023.1293342)

## Rarefaction curves

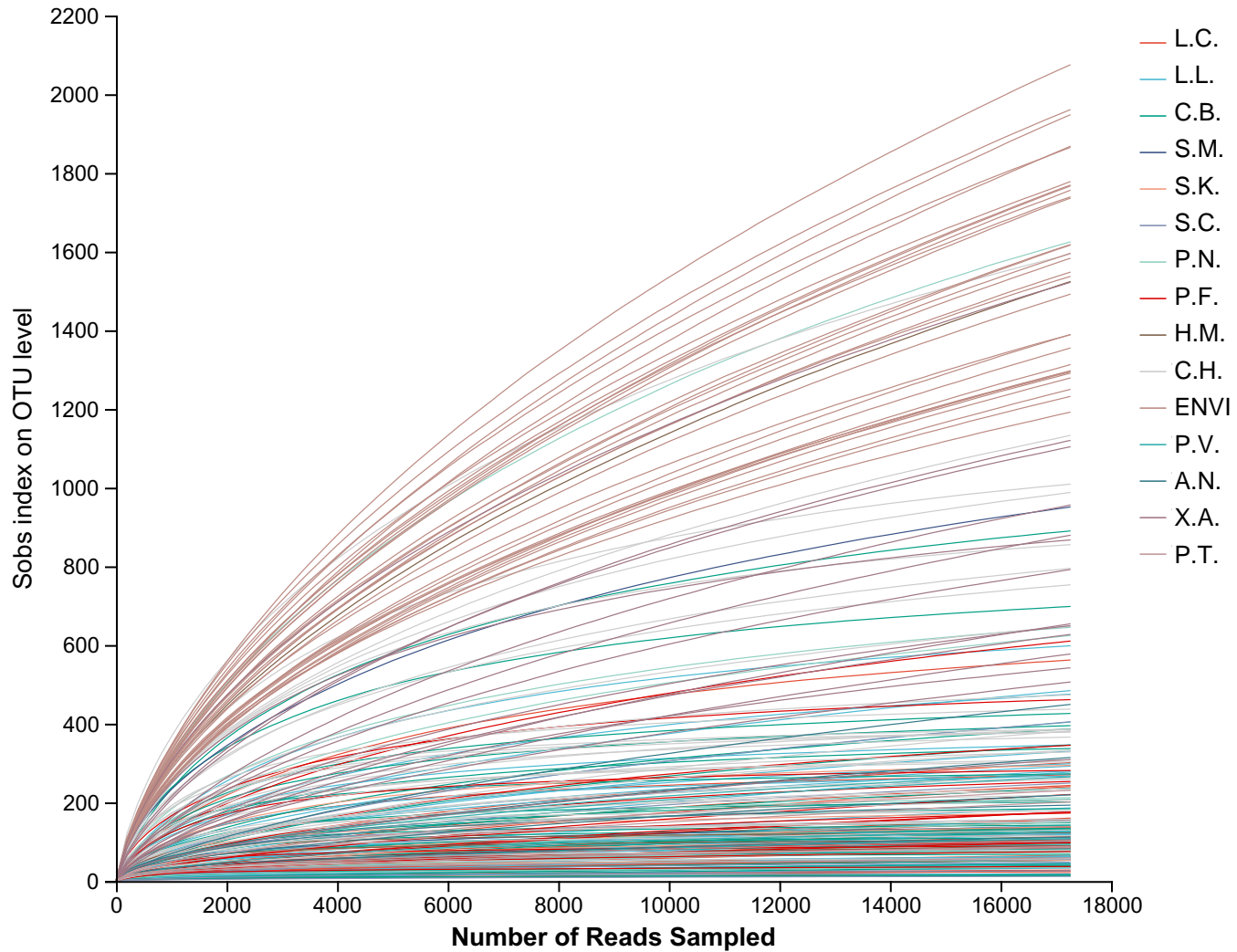

Supplement: Supplementary file 2 [file Data_Sheet_1.ZIP › Supplementary Figure S1 Rarefaction sobs.pdf]

# Potentially\_Pathogenic

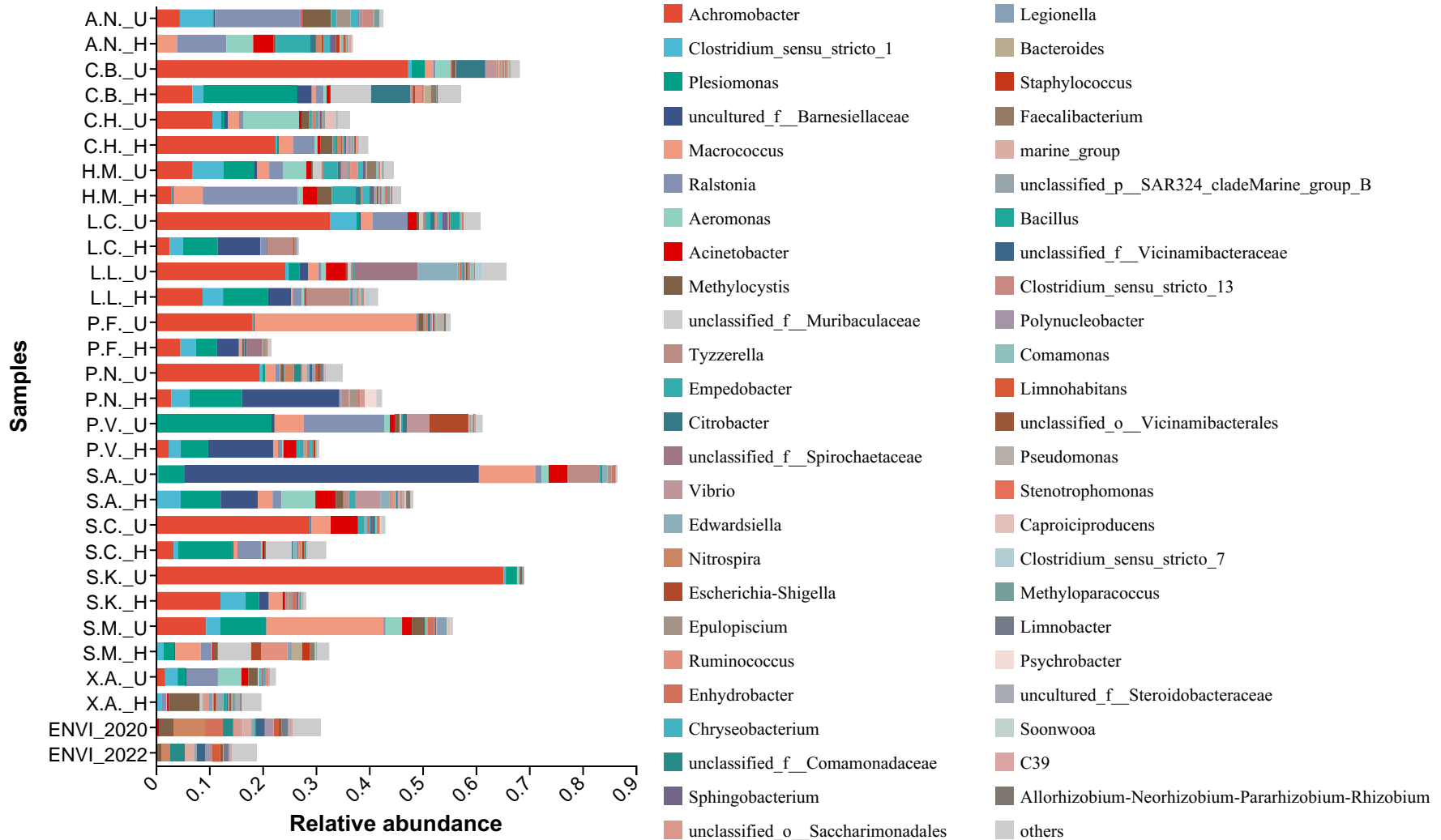

Supplement: Supplementary file 2 [file Data_Sheet_1.ZIP › Supplementary Figure S10 potential pathogenicity_g.pdf]

# Contains\_Mobile\_Elements

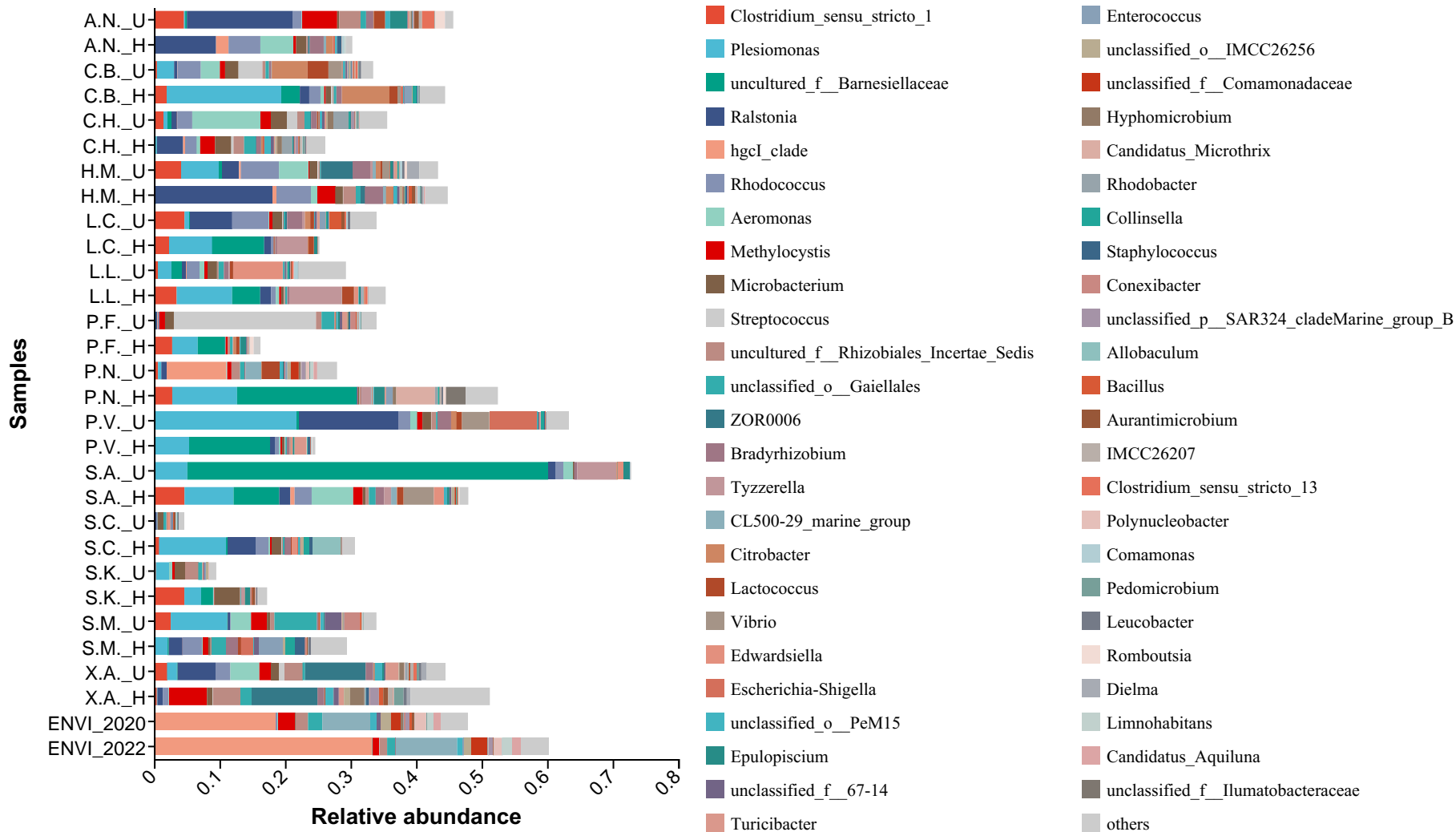

Supplement: Supplementary file 2 [file Data_Sheet_1.ZIP › Supplementary Figure S11 mobile element content_g.pdf]

# Stress\_Tolerant

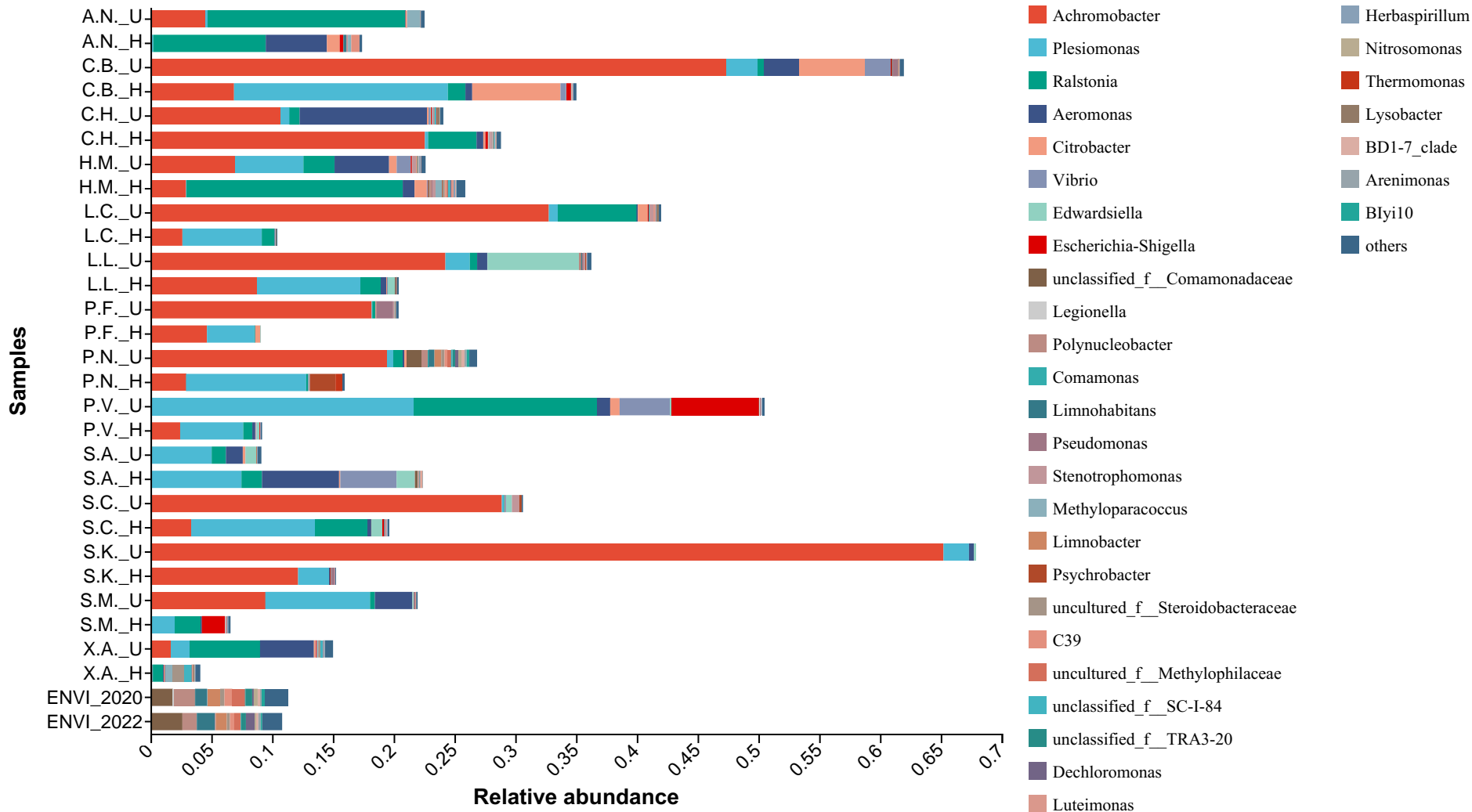

Supplement: Supplementary file 2 [file Data_Sheet_1.ZIP › Supplementary Figure S12 oxidative stress tolerance_g.pdf]

Cladogram

unhealthy omnivorous group  
healthy omnivorous group

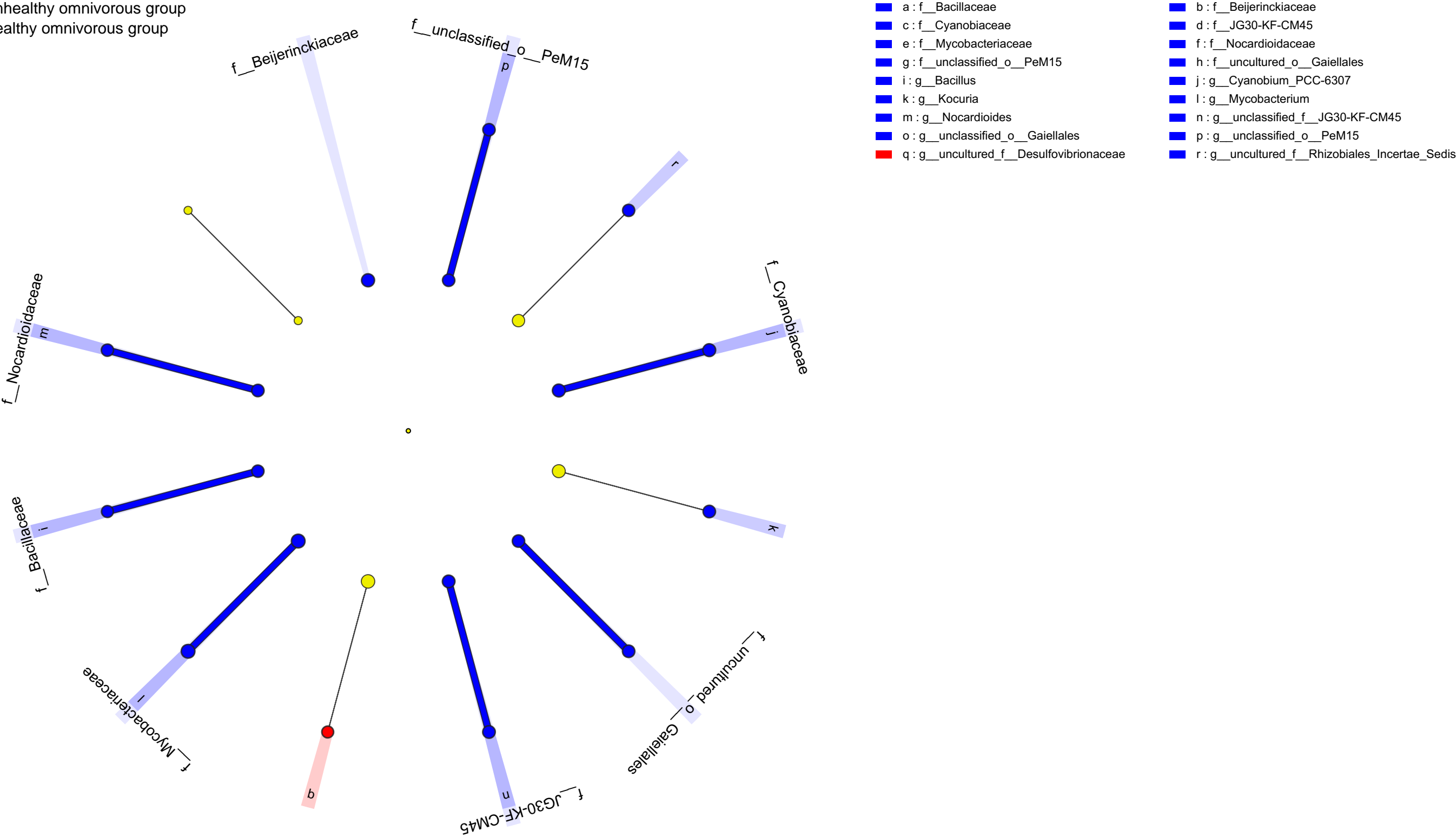

Supplement: Supplementary file 2 [file Data_Sheet_1.ZIP › Supplementary Figure S15 LDA omnivorous.pdf]

## Cladogram

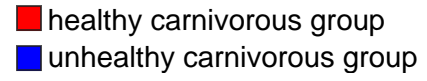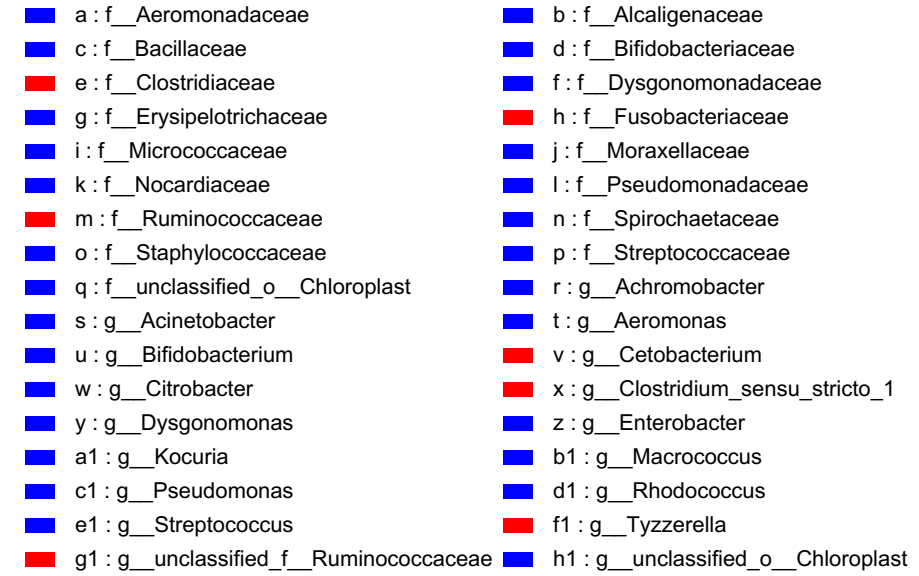

Supplement: Supplementary file 2 [file Data_Sheet_1.ZIP › Supplementary Figure S16 LDA carnivorous.pdf]

# Chao curves

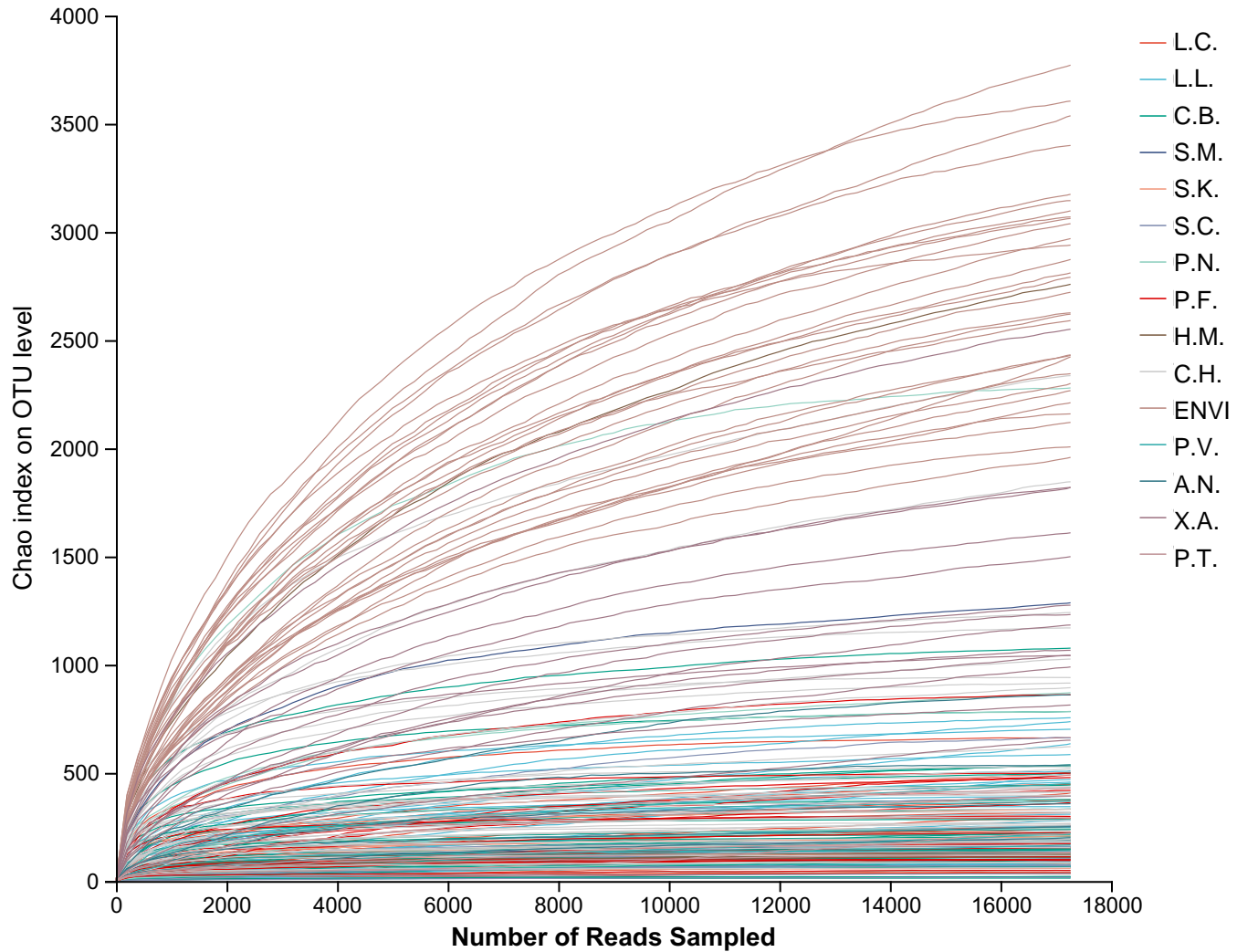

Supplement: Supplementary file 2 [file Data_Sheet_1.ZIP › Supplementary Figure S2 Rarefaction chaos.pdf]

Hierarchical clustering tree on Family level

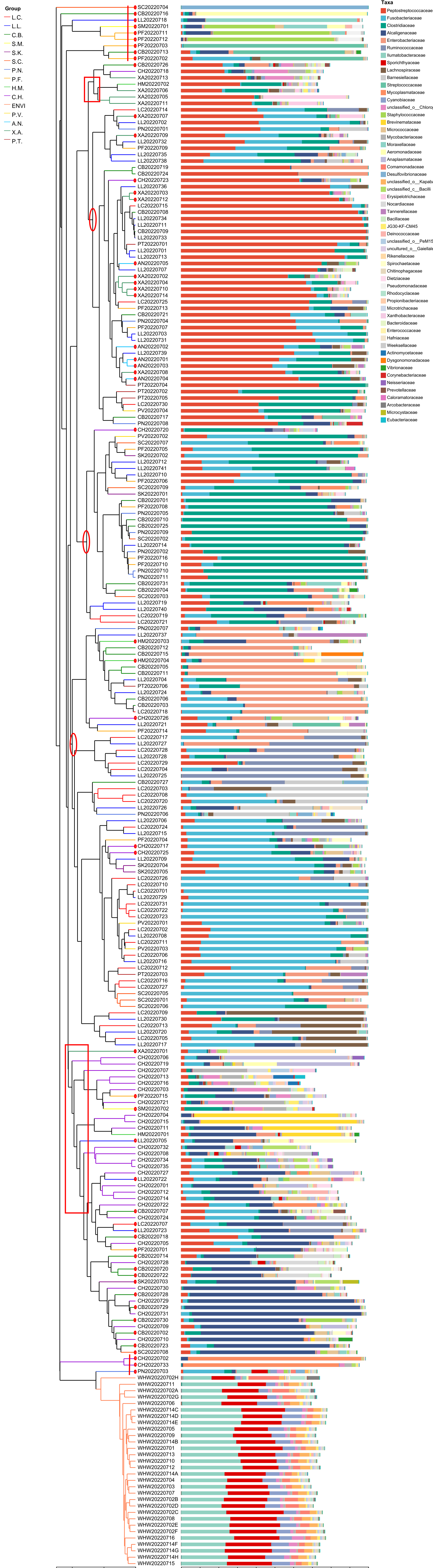

Supplement: Supplementary file 2 [file Data_Sheet_1.ZIP › Supplementary Figure S4 Hcluster family level.pdf]

Hierarchical clustering tree on Genus level

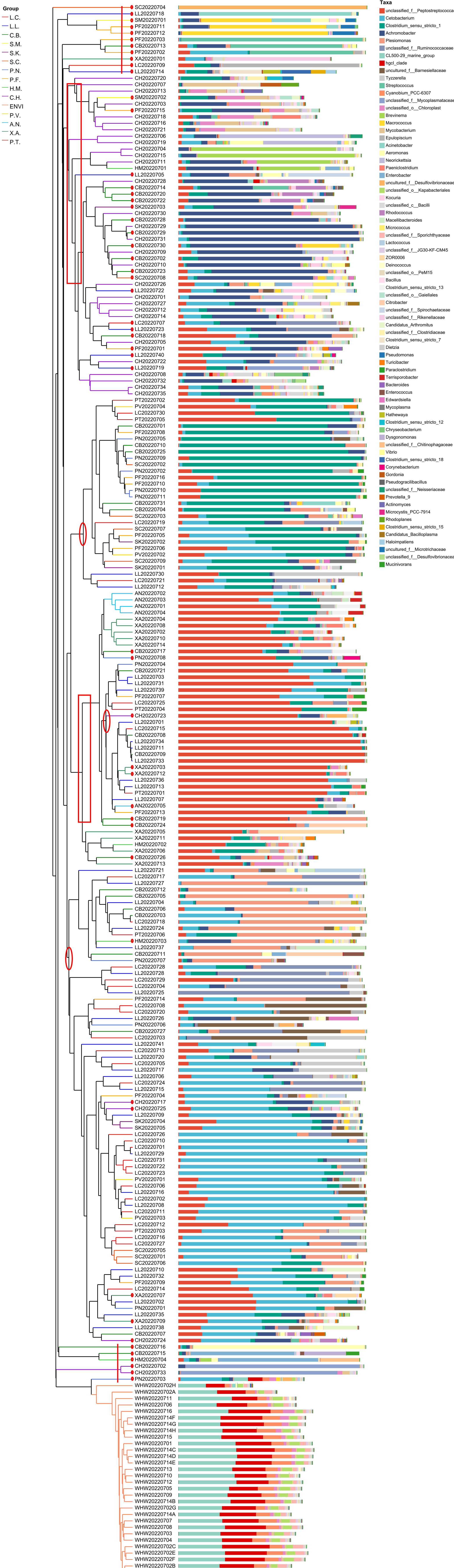

Supplement: Supplementary file 2 [file Data_Sheet_1.ZIP › Supplementary Figure S5 Hcluster genus level.pdf]

# Potentially\_Pathogenic

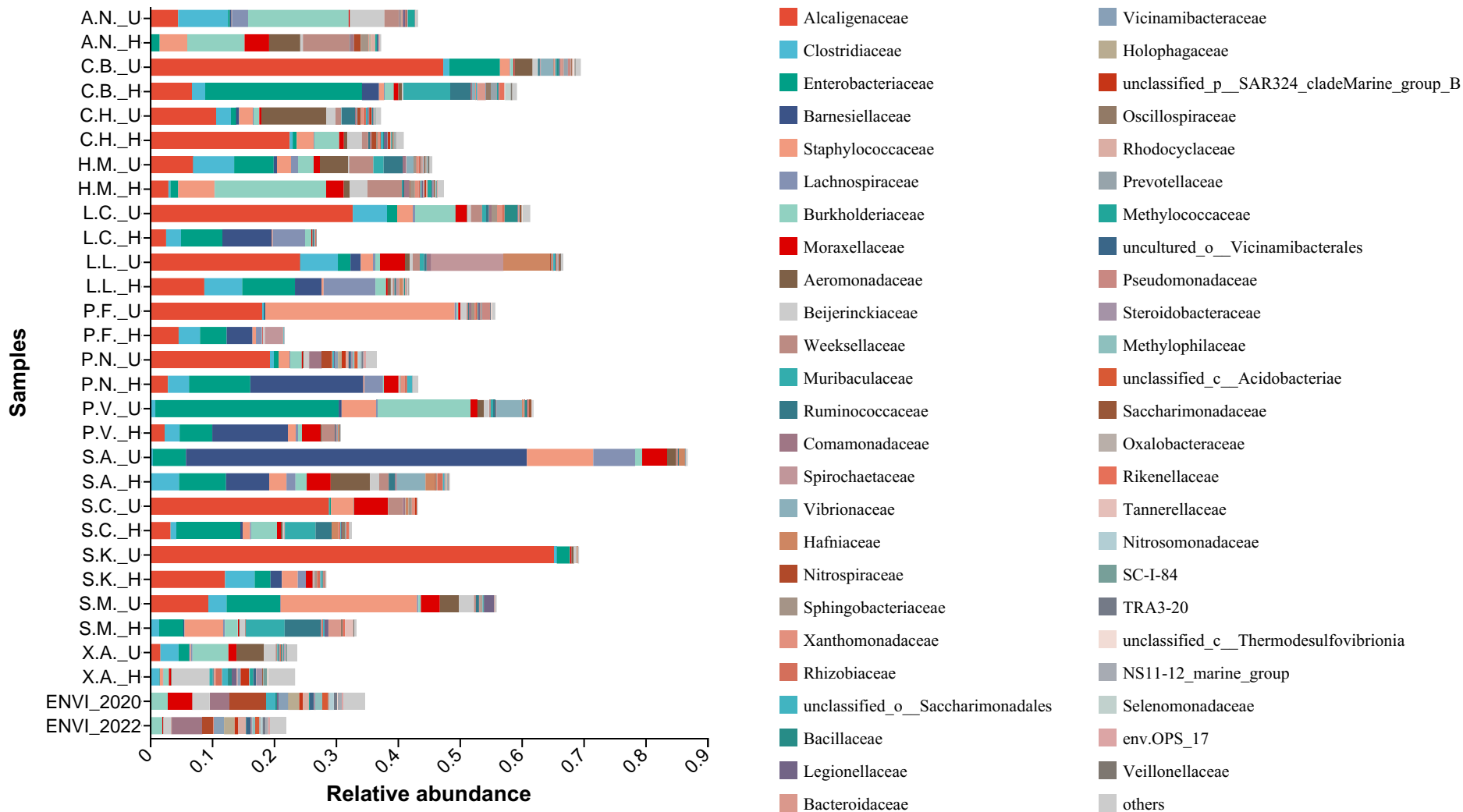

Supplement: Supplementary file 2 [file Data_Sheet_1.ZIP › Supplementary Figure S7 potential pathogenicity_f.pdf]

# Contains\_Mobile\_Elements

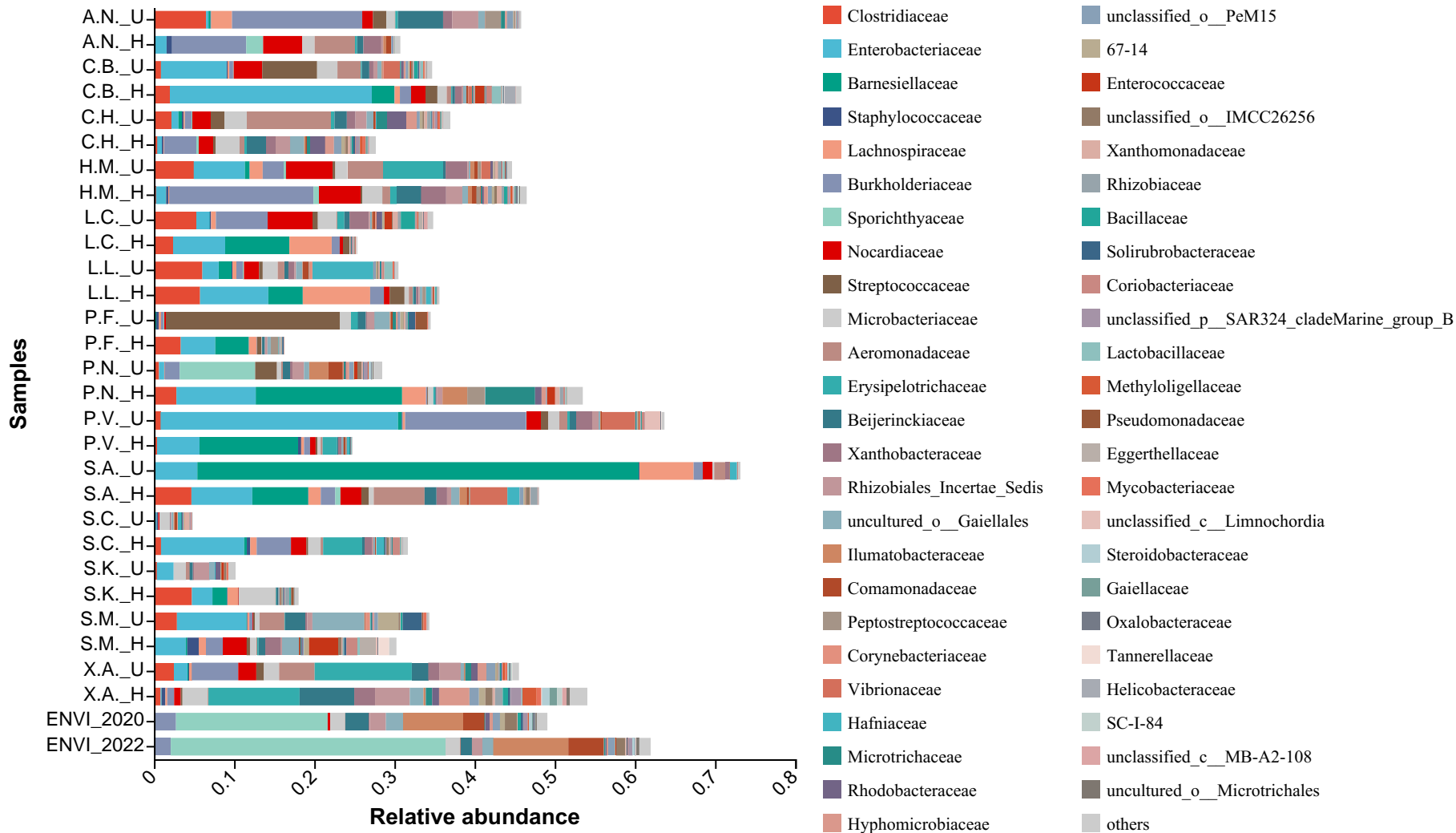

Supplement: Supplementary file 2 [file Data_Sheet_1.ZIP › Supplementary Figure S8 mobile element content_f.pdf]

# Stress\_Tolerant

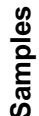

Supplement: Supplementary file 2 [file Data_Sheet_1.ZIP › Supplementary Figure S9 oxidative stress tolerance_f.pdf]
